# Primary Blast-Induced Traumatic Brain Injury as a Risk Factor for (Cerebro)vascular Disorder: Clinical Manifestations, Blast Physics, Biomechanics, Pathobiology, and Critical Gaps

**DOI:** 10.3390/ijms27114669

**Published:** 2026-05-22

**Authors:** Denes V. Agoston, James S. Meabon

**Affiliations:** 1 Department of Anatomy, Physiology and Genetic, School of Medicine, Uniformed Services University, Bethesda, MD 20814, USA; 2 Veterans Affairs Northwest Mental Illness Research, Education, and Clinical Center (MIRECC), VA Puget Sound Health Care System, Seattle, WA 98108, USA; james64@uw.edu; 3 Department of Psychiatry and Behavioral Sciences, University of Washington School of Medicine, Seattle, WA 98259, USA

**Keywords:** blast wave, acoustic impedance, vascular injury, endothelial stress, neuroinflammation, temporal evolution

## Abstract

Exposure to blast waves without kinetic, penetrating, thermal, or toxic components causes a distinct form of traumatic brain injury, termed primary blast-induced TBI (pbTBI). Clinical manifestations of pbTBI span a wide spectrum, ranging from life-threatening intracranial hemorrhage, hyperemia, and delayed cerebral edema to mild and transient neurological symptoms without detectable structural abnormalities on routine imaging. At the mild end of the spectrum, symptoms after a single exposure may resolve quickly, yet repeated exposures—even at very low levels, termed “subconcussive”—can develop into post-concussive syndrome (PCS) or persistent post-concussive symptoms (PPCS) in a subset of individuals. Despite extensive studies, the molecular pathobiology linking primary blast exposure to delayed and sometimes chronic neurobehavioral deficits remains incompletely understood. A mechanistic framework connecting blast-wave physics to biomechanics to biological vulnerability may therefore help define exposure hazards, interpret clinical symptomatology, and guide diagnostic and therapeutic development. This review summarizes the physics of primary blast waves, the resulting biomechanical responses, and candidate biological substrates, emphasizing structures and interfaces with distinct acoustic impedances across anatomical, tissue, cellular, and molecular scales. We synthesize evidence supporting the hypothesis that the cerebral vasculature and endothelial cells represent critically vulnerable substrates of primary blast-wave injury, in part because the vascular tree constitutes the brain’s largest and most widely distributed interface between compartments with different acoustic impedances. Across experimental and human studies, endothelial stress, vascular injury, and downstream neuroinflammation emerge as convergent molecular responses to primary blast exposure. Temporal dynamics are central to understanding pbTBI because many blast-induced processes unfold in sequential phases. These observations support conceptualizing pbTBI as a condition characterized by prominent cerebrovascular injury of varying severity with secondary consequences for neuronal signaling, network function, and behavior. Within this framework, cerebrovascular and neurovascular unit (NVU) dysfunction provides a parsimonious bridge between primary blast-wave exposure and chronic symptom trajectories, where vascular pathology may offer more accessible therapeutic targets than neuronal injury. Key knowledge gaps include identifying which physical component(s) of the blast are most injurious, establishing biologically meaningful dose–response relationships at molecular and physiological levels, and defining windows of vulnerability during recovery that are relevant to repeated exposures. Addressing these gaps is essential for refining safety protocols, improving diagnostic specificity through mechanism-informed biomarkers, and developing evidence-based molecular and vascular therapeutic targets for pbTBI-associated conditions. Progress will require integrating waveform-aware dosimetry with longitudinal physiological and molecular monitoring across both preclinical and human cohorts. Such integration offers a practical path toward translating blast physics into actionable medical guidance for prevention, triage, and recovery management.

## 1. Introduction

Traumatic brain injury (TBI) has been a persistent driver of military morbidity, but the dominant injury mechanisms have evolved alongside weapons technology and the environments in which explosions occur [1,2,3]. During recent conflicts, widespread use of improvised explosive devices and other high-energy munitions, coupled with improved body armor that reduces penetrating injuries, shifted the clinical landscape toward blast-associated neurotrauma [4,5]. Between 2000 and 2024, hundreds of thousands of U.S. service members were diagnosed with TBI, with a substantial fraction attributed to explosive blast exposure or combined blast/impact mechanisms [2,6]. During operations Enduring Freedom, Iraqi Freedom, and New Dawn (OEF/OIF/OND), estimates suggest that 5–35% of deployed personnel experienced TBI, with explosive blast contributing to the majority of cases. Most were classified as mild [7,8].

Explosions generate a composite injurious environment that includes the shock front and subsequent blast wind capable of accelerating bodies and objects, along with additional hazards such as fragments, burns, and toxic inhalants [9,10,11,12,13,14,15,16]. Primary blast TBI denotes injury attributable to the overpressure wave itself, recognizing that field conditions rarely provide perfect isolation of injury components and that classification often rests on incomplete exposure reconstruction [10,14,17,18,19,20]. Even in the absence of gross lesions, the steep pressure rise, short duration, and spectral characteristics of the primary wave can impose transient, supraphysiological stress on fluid-filled and biomechanically heterogeneous intracranial compartments [21]. This distinction matters because a pressure-defined exposure plausibly couples most directly to vascular and cerebrospinal fluid interfaces [22], whereas kinetic impacts primarily load the brain through bulk acceleration and tissue strain [23,24]. For blast exposure and pbTBI terminology see Box 1.

We first review the clinical symptom spectrum of pbTBI, with particular attention to the repeated low-level exposures common in training environments and to the temporal evolution of symptoms that define PCS and PPCS. We then summarize blast-wave physics and highlight why peak overpressure alone is an incomplete descriptor of intracranial hazard, particularly when reflections and confinement reshape the waveform in real-world settings. Next, we briefly discuss biomechanical response hypotheses, covering spalling, shear, cavitation, and nanoscale stress propagation, while emphasizing how acoustic impedance mismatches may localize mechanical energy at specific anatomical and cellular interfaces. Building on this mechanistic foundation, we survey candidate biological substrates across peripheral and central compartments, focusing on the neurovascular unit, perivascular spaces, and synaptic junctions as distributed interfaces with large impedance gradients.

While previous reviews have surveyed blast physics, biomechanics, or neuropathology in relative isolation, this review makes three distinct contributions. First, we present an integrated mechanistic framework that traces a continuous causal chain from waveform physics through acoustic impedance principles to specific biological substrates, rather than treating these as parallel but disconnected topics. Second, we explicitly advance and critically defend—rather than assume—the hypothesis that cerebrovascular and NVU dysfunction constitutes a primary pathobiological axis of pbTBI, engaging competing mechanisms and their evidentiary basis throughout, as synthesized in Table 1. Third, we translate identified knowledge gaps into practical experimental and clinical frameworks, providing actionable guidance for research design and clinical management that is absent from prior reviews in this space.

Box 1 **Definitions (blast exposure and pbTBI terminology).**

Definitions used throughout for explosive blast, blast-related TBI, and primary blast TBI (pbTBI).Explosive blast: Rapid energy release producing a transient blast wave (shock front, positive phase, often a subsequent negative phase), plus blast wind; real events may also include thermal effects and fragmentation/debris.Key measurements: Peak overpressure (Ppeak), impulse (pressure–time integral), positive-phase duration, rise time (dP/dt), and an energy proxy, such as TNT equivalent (when applicable).Propagation and environment: In open air, the wave expands outward; in enclosed/complex spaces, reflections and channeling can amplify and reshape local exposures.Effect taxonomy: Primary (blast wave), secondary (projectiles/debris), and tertiary (body displacement/impact) mechanisms often co-occur.Blast traumatic brain injury (bTBI): Brain injury arising from the complex exposure environment of blast, potentially combining primary, secondary, and tertiary components.Primary blast TBI (pbTBI): Injury attributable to the blast wave itself; clinically spans mild, moderate, and severe phenotypes.Mild vs. subconcussive pbTBI: Mild pbTBI typically corresponds to GCS 13–15; subconcussive exposure is GCS 15 without full concussion symptomatology but may still produce cellular/molecular injury.Repeated low-level blast: Recurrent mild/subconcussive exposures may yield cumulative risk and long-term structural and functional abnormalities.

## 2. Clinical Symptomatology of pbTBI and the Injury Spectrum

**Figure 1:**
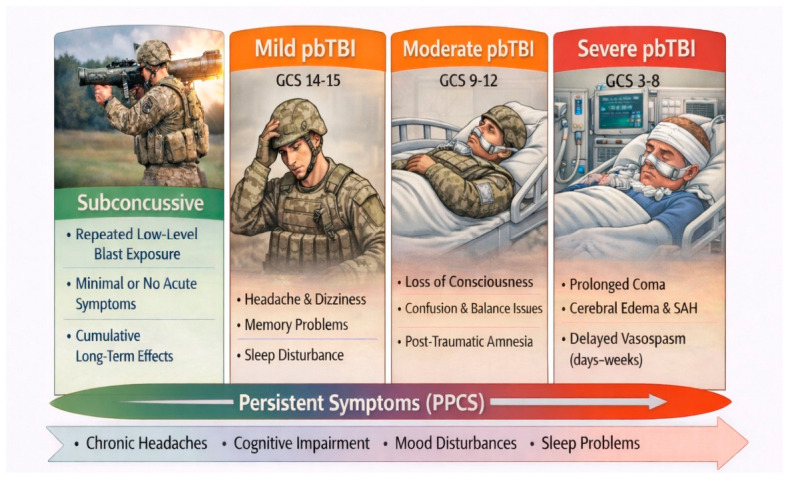
Spectrum of primary blast-related traumatic brain injury (pbTBI) and associated outcomes. Schematic illustration of the continuum of injury severity following exposure to explosive blast overpressure, ranging from subconcussive exposures to mild, moderate, and severe pbTBI. Subconcussive exposure may produce minimal or no acute symptoms but can have cumulative long-term effects. Mild pbTBI is associated with headache, dizziness, cognitive difficulties, and sleep disturbances. Moderate pbTBI involves prolonged alterations in consciousness, confusion, balance problems, and post-traumatic headaches. Severe pbTBI is characterized by high mortality risk (30–35%) and prolonged coma, with diffuse subarachnoid hemorrhage and rapid cerebral edema. Some individuals develop persistent post-concussive symptoms (PPCS), including chronic headaches, cognitive impairment, mood disturbances, and sleep disruption lasting months to years. The figures were created using ChatGPT Version 1.2026.048 (1771630681).

Depending on the intensity of explosion and environmental factors that modify its effect—as discussed later—the clinical manifestations, i.e., the severity of pbTBI, range across the entire TBI injury spectrum (Figure 1). The spectrum representation in Figure 1 is intended to convey the continuous rather than categorical nature of blast injury severity, with symptom burden and recovery trajectory depending heavily on exposure characteristics and individual vulnerability. See also Box 2 and Box 3. At the severe end of the pbTBI spectrum, patients may present with profound neurological compromise, including Glasgow Coma Scale scores in the 3–8 range, prolonged unconsciousness, and extended post-traumatic amnesia [25]. The mortality rate for severe pbTBI is high—30–35% in an acute setting [26,27]. Operational experience during OEF/OIF/OND highlighted a phenotype marked by very rapid onset cerebral edema, frequently within an hour, often accompanied by diffuse subarachnoid hemorrhage and hyperemia that underscored a vascularly centered catastrophe rather than focal contusion [28,29,30,31,32,33]. A distinctive and clinically consequential feature described in severe cases is delayed-onset malignant vasospasm occurring days to weeks after exposure, which drives late neurological deterioration and mortality [34]). Although vasospasm has been hypothesized as compensatory in the setting of dysregulated cerebral blood flow, its initiating mechanisms and relationship to blast-specific vascular signaling remain incompletely defined [35].

Box 2 **Structured Blast Exposure and Symptom Questionnaire.**

Domain A—Exposure Characterization: Distance from blast source (estimated, meters); indoor/outdoor/vehicle-confined environment; blast count and inter-exposure interval in the preceding 72 h; longest blast-free interval in the preceding 30 days; career cumulative exposure estimate; protective equipment used (helmet type, hearing protection, and body armor).

Domain B—Acute Symptom Onset and Temporal Profile (0–72 h): Time from blast to first symptom; symptom trajectory over 48 h (improving/stable/worsening); tinnitus and hearing loss (blast-specific discriminators); amnesia type and duration (retrograde/anterograde); loss of consciousness (duration if present). Domain C—Subacute and Chronic Symptom Surveillance (0–90 days): Headache characteristics (frequency, severity, and location); sleep disruption; vestibular and autonomic symptoms; cognitive complaints (attention, memory, and processing speed); mood and affective regulation; endocrine screen sensitive to post-traumatic hypopituitarism (disproportionate fatigue, thermoregulatory symptoms, and libido changes).

Box 3 **Guiding Principles for Clinicians Managing pbTBI.**

1. Treat blast-associated TBI as mechanistically distinct from kinetic TBI for purposes of return-to-duty decisions; do not apply kinetic-TBI symptom resolution timelines to blast exposures.

2. Prioritize exposure characterization beyond peak overpressure: record distance, environment (open-field vs. confined), blast count, and inter-exposure interval prospectively.

3. Apply conservative inter-exposure vulnerability windows given the absence of human interval data; err toward longer rest periods when cumulative exposure history is unknown.

4. Systematically screen for vestibular, auditory, and endocrine sequelae that distinguish blast from kinetic-TBI phenotypes: tympanometry, pure-tone audiogram, growth hormone and IGF-I axes.

5. Perform differential diagnosis of pbTBI from PTSD at intake using validated instruments; symptom overlap is high and misclassification has management consequences.

6. Monitor for delayed deterioration beyond acute stabilization: vasospasm (days to weeks) and biphasic BBB disruption (subacute inflammatory phase) may require re-evaluation.

7. Prospectively document cumulative blast exposure histories across the career; baseline biomarker levels in blast-experienced personnel may differ substantially from unexposed norms.

Moderate pbTBI typically involves Glasgow Coma Scale scores in the 9–12 range, with altered consciousness up to 24 h, post-traumatic amnesia up to seven days, and persistent confusion, disorientation, and executive dysfunction that can last days to weeks [32]. While symptom domains overlap with moderate kinetic TBI, blast-associated presentations more often include tympanic membrane rupture, tinnitus, and vestibular dysfunction that can limit return-to-duty even when cognitive measures improve [11,19]. Recovery trajectories are frequently heterogeneous, with many individuals improving over weeks to months, but a substantial subset developing lasting symptoms [36,37,38]. These symptoms resemble post-concussive syndrome (PCS) and/or persistent post-concussive syndrome (PPCS) characterized by mood lability, irritability, and impaired emotional regulation [36]. This heterogeneity is often interpreted through the lens of mixed mechanisms because moderate blast exposures may include both primary wave loading and secondary acceleration and/or stressors that complicate attribution [37,38,39].

PCS and PPCS represent symptom constellations defined by persistence of somatic, cognitive, and affective symptoms beyond the expected acute recovery period, chief among which are headache, sleep disruption, vestibular dysfunction, attention and memory deficits, executive dysfunction, and sensory sensitivities [40,41,42,43,44]. By convention, PCS encompasses persistence up to roughly three months and PPCS refers to persistence beyond three months, sometimes for years [45], with potentially substantial impacts on occupational performance and quality of life [46]. The pathophysiology remains debated, but neuroinflammatory mechanisms, microvascular dysregulation, and impaired cerebrovascular reserve are well-supported mechanisms that can produce functional impairment without gross lesions [47,48]. Diagnostic complexity arises because overlapping symptom domains can also occur with PTSD and other stress-related conditions, making differential diagnosis challenging and underscoring the need for mechanistically informative biomarkers [49,50,51,52].

Mild pbTBI, commonly defined by a Glasgow Coma Scale of 14–15 and transient neurobehavioral alteration without external injury or routine imaging abnormalities [53], constitutes the most prevalent clinical presentation in both combat and training contexts [6,11,20,54,55]. Acute symptoms within 72 h frequently include headache, dizziness, confusion, memory impairment, balance problems, nausea, irritability, and concentration deficits, with headache and sleep disturbance among the most common complaints [44,56,57,58,59,60]. Although this symptom set overlaps strongly with both non-blast mild TBI and PTSD, auditory complaints including hearing loss and tinnitus tend to be more prominent after blast exposure, consistent with parallel tympanic and vestibular loading [25,61,62]. Most individuals report symptom resolution within days, which supports rapid return-to-duty decisions but also increases the likelihood of re-exposure during the period of increased cerebral vulnerability [63,64,65] that can adversely affect the recovery process. As a result, repetitive blast exposure within shorter intervals, even when at low overpressures during weapons system training, can be expected to increase symptom burden and persistence and or precipitate delayed symptom development [66,67].

Repeated low-level “subconcussive” blast exposures during weapons system use create an exposure pattern that differs from single-incident mTBI/concussion paradigms and is therefore poorly captured by traditional clinical frameworks [55]. Occupational roles such as range safety officers can involve thousands of exposures across a tour, and these personnel often report cognitive and behavioral symptoms that are subtle at rest but emerge with sustained demand, sleep disruption, and (presumably) age-related decline [68,69,70,71,72,73,74,75,76]. Experimental studies and emerging training-based cohorts suggest that cumulative effects depend not only on exposure count but also on exposure density, implying a recovery-dependent vulnerability window analogous in principle, though not necessarily in mechanism, to “second impact” concepts described in kinetic TBI [63,64,65]. Because most human studies lack precise interval recording, the field cannot yet specify safe inter-exposure windows, nor can it distinguish transient adaptation [77] from progressive dysfunction in individuals with high exposure density [12,78].

## 3. Clinical Limitations

**Figure 2:**
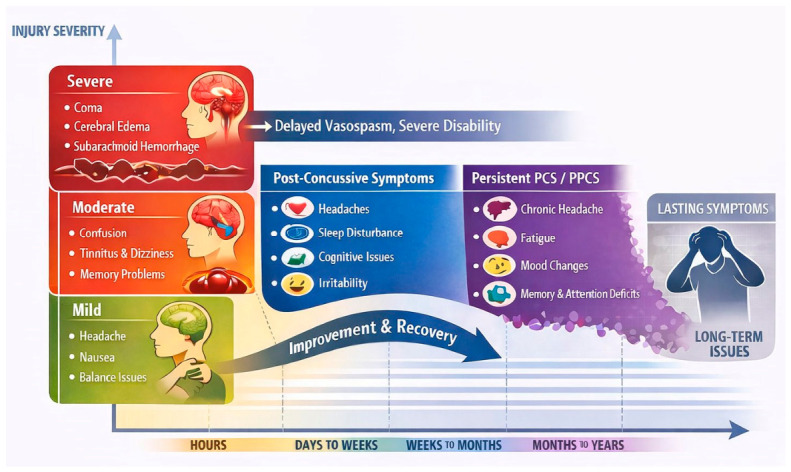
Temporal evolution of symptoms following primary blast-related traumatic brain injury (pbTBI). Diagram illustrating the relationship between injury severity and the temporal progression of clinical manifestations. Mild injury is associated with acute symptoms (headache, nausea, and balance disturbances) that often improve. Moderate injury may produce confusion, tinnitus, dizziness, and memory problems, with post-concussive symptoms in the subacute phase. Severe pbTBI can result in coma, cerebral edema, and subarachnoid hemorrhage, with potential for delayed vasospasm and severe disability. A subset develops persistent post-concussive symptoms (PPCS), including chronic headaches, fatigue, mood changes, and memory and attention deficits lasting months to years. The figures were created using ChatGPT Version 1.2026.048 (1771630681).

Several limitations constrain how current clinical data are interpreted and how pbTBI is classified in both combat and training environments. (See Box 2 and Box 3). First, attribution of blast-related symptoms to primary wave effects is often uncertain because real-world events rarely isolate mechanisms, exposure reconstruction is incomplete, and environmental complexity can obscure whether secondary or tertiary components contributed [10,79]. Second, most studies of repeated exposures, which represents the scenario of greatest relevance to occupational health, lack precise recording of inter-exposure intervals, leaving the duration of post-blast cerebral vulnerability largely unknown (Figure 2). Third, comorbidities and co-exposures, including prior concussions and symptom overlap with PTSD [50,52,80,81,82,83], complicate differential diagnosis and can lead to misclassification or inflated associations when symptom-based endpoints are used without mechanistic biomarkers [12,18,84].

## 4. Physics of Explosive Blast and Its Components

Explosive blast is best conceptualized as a transient energy wave [85,86,87]. For the definition of primary blast see Box 4.

The wave propagates through air and, upon encountering objects and tissues, undergoes reflection, refraction, absorption, and mode conversion, which can reshape the delivered waveform [88,89,90]. In idealized open-field conditions, blast waves are often approximated by a Friedlander waveform, characterized by an abrupt shock-front arrival, a near-instantaneous rise from ambient pressure to a peak overpressure, a short positive phase, and a longer negative phase of sub-ambient pressure before gradual return to baseline [91]. The shock front is followed by the blast wind, a slower-moving flow component that can impart momentum to bodies and objects and thereby introduce tertiary acceleration mechanisms that may coexist with primary wave effects [10,17,92]. Real-world exposures are rarely idealized and are instead shaped by complex environmental interactions [88,89,90]. Because the brain’s response depends as much on the temporal course of pressure loading as on peak magnitude, environmental complexity can significantly alter tissue loading in ways that remain understudied.

Box 4 **Physics (primary blast exposure).**

Primary blast physics defines the external loading inputs that initiate tissue-level injury.A blast wave is a transient pressure disturbance with a sharp shock front, positive phase, and often a subsequent negative phase; the waveform is reshaped by reflection, confinement and material properties.Dose parameters include peak overpressure (PSI, kPa), positive-phase duration (millisecond scale), impulse (pressure-time integral), rise time (dP/dt), and spectral content.Environment matters, as distance, orientation, shielding, and enclosure geometry can modify exposure via attenuation, reflections, and/or complex wave interactions.Primary blast (wave) is distinct from secondary (debris) and tertiary (body acceleration/impact) mechanisms, which often co-occur in real events.

Peak overpressure remains the most commonly recorded metric in both operational settings and experimental models, but it captures only one dimension of blast physics and can therefore misrepresent biological risk [10,93]. Impulse, which integrates pressure over time, relates more directly to momentum transfer and may help explain why exposures with similar peaks can have different physiological consequences. The negative pressure phase is frequently underemphasized despite its potential relevance to cavitation in fluids because tensile components can lower local pressures below nucleation thresholds in cerebrospinal fluid and intravascular compartments [94,95,96,97,98,99]. In addition, rise time can be decisive for microvascular loading, because steeper fronts introduce higher-frequency content that preferentially stresses small-scale impedance discontinuities and boundary layers.

Spectral characteristics of blast waves provide a third, often-neglected dimension [100]. Explosive blast generates complex energy waves comprising varying amplitudes and frequencies, ranging from Hz to sub kHz to kHz [16,92,101]. Blast-wave frequency bands typically concentrate around 10–50 Hz, and with increasing distance from the blast center, the main frequency diminishes following a power law and frequency bands become narrower [100]. The relative distribution depends on charge size, distance, environmental reflections, weather conditions, and the material properties of surrounding structures [102]. As distance from the blast source increases, dominant frequencies tend to shift lower and the bandwidth narrows, yet in confined spaces reflections can sustain higher-frequency components that would otherwise dissipate. This feature is mechanistically important because biological structures are frequency-selective mechanical filters [79,103,104]. From a tissue-mechanics standpoint, lower-frequency, higher-amplitude components can promote bulk motion and shear, whereas higher-frequency components can concentrate stress at sharp impedance transitions, such as skull–CSF, CSF–parenchyma, and vascular–parenchymal interfaces [21,103,104,105]. These physical complexities create three practical limitations for the field that directly influence how we interpret clinical and experimental findings. First, current “dose” conventions often rely on peak pressure thresholds that were historically derived from non-neurological endpoints such as tympanic membrane rupture probabilities [106]. This may not map cleanly onto brain injury mechanisms involving prominent impedance barriers, such as gray–white matter interfaces or the NVU [21,22,107]. Second, sensor-based dosimetry is inherently variable because incident and reflected pressures differ substantially at body surfaces, helmet-mounted sensors can over- or under-estimate intracranial loading, and orientation relative to the wave can dominate measured values even when exposures appear similar [108,109]. Third, retrospective reconstruction is difficult because many studies lack sufficient detail to model local pressure fields without site-specific environmental data [16,92,101]. The physical parameters and their biological relevance are summarized in Table 1 (physics rows).

## 5. Biomechanics of Primary Explosive Blast

The biomechanical response of the head to primary blast emerges from an interaction between waveform properties and the structural and material complexity of the skull and intracranial compartments [24,110,111,112]. For the definitions of biomechanics blast, see Box 5. Because blast waves contain both low- and high-frequency components, the skull and scalp can act as a frequency-dependent filter that attenuates some components while transmitting others into intracranial fluids and tissues [103,113]. This filtering matters because the brain is not a homogeneous solid but rather a fluid-saturated, viscoelastic composite with large impedance discontinuities at skull–CSF, CSF–parenchyma, blood–vessel wall, and gray–white matter interfaces [114,115,116,117,118,119]. Accordingly, the same nominal external pressure can generate very different internal stress fields depending on orientation, confinement, and the degree to which intracranial fluids are driven into oscillation. The canonical candidate mechanisms, including spalling, shear, cavitation, and nanoscale strain propagation, can be understood as different manifestations of energy localization at these boundaries and within fluids rather than mutually exclusive explanations.

Box 5 **Biomechanics (coupling of blast to head and brain).**

Biomechanics links blast-wave descriptors to the internal deformation fields that drive biological damage.Acoustic impedance mismatches (skull, CSF, parenchyma, and blood) govern reflection/transmission and can concentrate stress at interfaces.Injury-load paths include rapid intracranial/cerebrovascular pressure gradients, bulk or focal deformation, and shear at gray–white and perivascular boundaries.Waveform features (impulse, duration, rise time, and frequency content) can differentially weight pressure-driven vs. shear-driven components.Multi-mechanistic consideration: primary blast may injure without direct head impact, but concurrent acceleration/impact (tertiary) can add classic concussion biomechanics.

These four candidate mechanisms—shear, spalling, cavitation, and nanoscale strain propagation—are not equally supported by empirical evidence and do not contribute uniformly across blast intensities. Shear-related white matter injury has the strongest translational support, drawing from human diffusion tensor imaging (DTI) cohort studies and a mature kinetic-TBI literature. Spalling is well-grounded in acoustic impedance physics and consistent with postmortem neuropathological distributions at impedance boundaries, but direct in vivo human validation remains absent; the supporting human evidence is primarily indirect and autopsy-based. Cavitation is mechanistically compelling—particularly for explaining vascular boundary pathology—yet human intracranial pressure thresholds have not been established and prevention may prove extremely difficult under normal physiological conditions. Nanoscale strain propagation is the least empirically validated of the four, resting primarily on computational modeling with no direct in vivo corroboration in blast-exposed biological tissue. Intensity-dependence likely demarcates when each mechanism dominates: bulk shear and spalling may predominate at moderate-to-high blast levels, while cavitation-related and nanoscale mechanisms may become relatively more prominent at lower intensities where gross deformation is absent.

Acoustic impedance provides a compact principle for predicting where blast energy is likely to be reflected, transmitted, and deposited within the head. Acoustic impedance is determined by tissue density and wave propagation speed [120,121], and it differs dramatically across biological materials, with bone exhibiting far higher impedance than soft tissues and with air representing an extreme mismatch relative to both [21]. When a pressure wave encounters an impedance boundary, part of the wave reflects and part transmits, and the reflected component can invert into tensile loading depending on the boundary conditions, creating opportunities for microstructural failure. These principles parallel those exploited in extracorporeal shock wave therapies and therapeutic ultrasound, where energy deposition is intentionally focused at impedance boundaries to fragment targets or stimulate tissue responses [122,123]. In pbTBI, the implication is not that the brain experiences “ultrasound” per se but that brief blast-induced loading can deposit energy preferentially at interfaces of impedance mismatch where the biological cost of barrier disruption is high [21].

Spalling refers to internal failure that occurs when a compressive wave encounters an impedance discontinuity and reflects as a tensile wave whose magnitude exceeds local material strength [17,124,125,126]. In the brain, spalling sites include the skull–brain boundary, CSF–parenchyma interfaces, gray–white matter junctions, and blood vessel–parenchyma interfaces, all of which combine mechanical contrast with biological specialization [21]. At these locations, microfailures could manifest as microscopic tearing, endothelial junction disruption, or perivascular microlesions that are not detectable on conventional imaging yet can alter vascular permeability and cellular signaling. Because spalling is boundary-driven, it provides a mechanistic explanation for why neuropathology can cluster near vessels, ventricular walls, and subpial surfaces even when the parenchyma appears grossly intact [10,127,128]. Spalling is well-supported by acoustic impedance physics and consistent with postmortem findings of neuropathology clustering near vessels, ventricular walls, and subpial surfaces, but direct in vivo human imaging validation is absent; the supporting human evidence is indirect and autopsy-based. It is most plausibly operative at high blast intensities.

Shear stress arises when adjacent tissue regions experience different accelerations or deformation rates, producing sliding forces that can disrupt axonal bundles, vascular structures, and cell–cell junctions even in the absence of macroscopic tearing [129,130,131]. Low-frequency components of the blast waveform can drive bulk motion and create shear at interfaces where gray matter, white matter, and fluid-filled spaces respond differently to the same pressure transient. Within the microvasculature, abnormal shear can trigger endothelial activation, cytoskeletal remodeling, and tight-junction reorganization through mechanotransduction pathways, driving endothelial stress responses. At the tissue level, shear can drive diffuse, spatially heterogeneous injury patterns seen in imaging studies, as shear strain fields can vary with local anatomy, gyral geometry, and ventricular proximity [128,132,133]. Shear has the strongest translational evidence base of the four candidate mechanisms: multiple human DTI cohort studies replicate white matter microstructural changes in blast-exposed populations, and the biomechanical mechanism is supported by a large kinetic-TBI literature. Shear is most plausibly operative at moderate-to-high blast intensities.

Cavitation describes the formation and collapse of gas or vapor bubbles in fluids exposed to rapid pressure changes [98,99]. During the negative phase of the blast waveform, transient pressures may fall below nucleation thresholds in CSF or intravascular fluids, enabling bubble formation that is subsequently driven to collapse as pressure re-equilibrates. The process can generate localized high-pressure spikes that load adjacent structures, including endothelium, basement membranes, and perivascular astrocytic endfeet, at spatial scales far smaller than those addressed by bulk strain metrics [98,134,135]. Computational models have suggested that cavitation can occur in sulci and periventricular regions and that higher-intensity exposures could extend cavitation into intravascular compartments, thereby directly stressing the NVU [94,96,132,136]. Although thresholds for in vivo cavitation in human intracranial fluids remain debated, this hypothesis is attractive because it explains how tensile physical waveforms result in the oft-reported boundary-localized pathology at vascular and CSF interfaces. However, even if cavitation-dependent injury mechanisms are confirmed, prevention may prove extremely difficult, as very little can stop bubble formation during the rapid negative pressure phase under conditions of normal human physiology. Direct in vivo validation of intracranial cavitation in humans is lacking, and human skull geometry, CSF volume, and intravascular gas saturation differ sufficiently from the rodent preparations and computational geometries used to generate these predictions that threshold estimates cannot be applied to human exposure scenarios without further validation.

Implosion effects compress liquid media, reducing local pressure sufficiently to nucleate dissolved gases into expanding bubbles within the CSF and cerebral blood [137,138]. Bubble formation and collapse follow a dual-wave mechanism: outer skull pressure waves generate cavitation bubbles, which are then driven to collapse by inner tissue pressure waves, releasing micro-jets and localized pressure spikes at CSF–brain and blood–brain interfaces. This mechanism provides a compelling explanation for the distinctive perivascular astroglial scarring observed in post-mortem brains of individuals who sustained bTBI or repeated bTBI exposures attributed to primary blast [128,139]. This mechanism has not been demonstrated in vivo. The predicted pressure-wave intervals and spatial scales derive from theoretical models that have not been validated by ultrastructural studies in blast-exposed biological tissue.

Nanoscale tissue injury is caused by shock waves passing through fluid compartments generating acoustic waves that in response can cause ultrastructural damage within microseconds [140]. Models predict periodic “nano” pressure waves at ~200 nanometer intervals with pressure peaks at ~4 nanometers, potentially affecting subcellular structures, such as intercellular connections and synaptic junctions, without causing detectable light microscopic level changes [140,141,142]. Nanoscale strain propagation is the least empirically validated mechanism and is most plausibly operative at subconcussive intensities where gross deformation is absent and molecular-scale perturbations may dominate.

Biomechanical inference in pbTBI is limited by three recurring issues that shape how experimental results translate to humans. First, much of the mechanistic literature borrows from materials science, ex vivo preparations, or computational modeling, which are essential but may not capture the coupled fluid–solid dynamics and physiological boundary conditions of the living human head [94,143,144,145,146]. Second, translatability across species is constrained by anatomical differences, including lissencephalic rodent brains, scaling of skull thickness, and differences in CSF compartment geometry, all of which can alter internal wave propagation and cavitation propensity [147,148]. Third, candidate mechanisms may be intensity-dependent, such that skull flexure and large-scale CSF driving may dominate at higher blast levels whereas low-level exposures preferentially perturb molecular and junctional substrates without overt deformation [149,150,151,152,153]. The biomechanical mechanisms and their translational status are summarized in Table 1 (biomechanics rows) and Table 2 (evidentiary tier summary).

## 6. Biological Substrates of pbTBI

Primary blast exposure is a whole-body event, and the pattern of biological substrates engaged by a given blast depends on the waveform characteristics, distance, and orientation, environmental reflections, and the modifying effects of helmets and body armor [112,154,155]. Because different tissues and organs present different acoustic impedances and fluid contents, they respond to the same blast with different modes of deformation, energy absorption, and potential injury [21,156,157]. This heterogeneity means that pbTBI cannot be reduced to a single “brain-only” mechanism, even when clinical presentation is primarily neurological, because peripheral organ responses can influence vascular tone, inflammatory signaling, metabolic reserve, and neurological function and behavior [158,159,160]. At the same time, within the cranial vault, the brain contains layered membranes, fluid spaces, and vascular networks, which create a dense landscape of impedance discontinuities where blast energy can concentrate [161]. For the definition of biological substrates of pbTBI, see Box 6.

Box 6 **Biological substrates of pbTBI.**

pbTBI neuropathology often yields microvascular and microstructural injury rather than large focal contusions.Vascular and neurovascular unit (NVU) vulnerability, endothelial stress, barrier disruption, and perivascular microlesions can occur with subtle gross pathology.Interface-enriched injury patterns are consistent with impedance-driven localization (e.g., perivascular, ventricular, and gray–white matter boundaries).White-matter microstructural injury may manifest as axonal/myelin alterations detectable by diffusion metrics even when conventional imaging is normal.Secondary cascades include neuroinflammation, oxidative stress, and impaired clearance pathways (e.g., glymphatic/perivascular space changes) that evolve over time.

Peripheral anatomical structures, particularly the thorax and abdomen, are vulnerable to blast overpressure and can generate systemic physiological consequences that may secondarily influence the brain [158,162,163]. Compression of the thorax and abdomen can produce rapid hemodynamic transients and hydrodynamic pulses that propagate through the vasculature, potentially stressing endothelial junctions and altering cerebral perfusion [162,164,165]. However, the extent to which these mechanisms contribute to pbTBI likely depends on the blast intensity and on whether the exposure includes substantial blast-wind components that load the body beyond the pure wavefront [166,167,168]. Pulmonary blast injury (commonly termed “blast lung”) illustrates how energy dissipation at air–tissue and tissue–blood interfaces can produce vascular-centered pathology and systemic inflammatory responses that could amplify neuroinflammation and worsen outcomes [169,170,171]. Because abdominal and pulmonary contributions to intracranial pathobiology remain understudied, they represent an important confounder and opportunity, as clarifying when these peripheral mechanisms dominate would sharpen the mechanistic specificity of pbTBI models.

Within the head, heterogeneity begins with the distinction between viscerocranium and neurocranium, because the facial skeleton contains multiple air-filled cavities whose impedance contrasts and local reflections differ from the cranial vault [137,172]. High-level blasts can produce maxillofacial injury through combined implosion–explosion phases that fracture bone and disrupt mucosal surfaces, whereas lower-level exposures may produce more subtle mucosal bleeding and inflammatory responses [137,173]. These injuries can plausibly influence brain outcomes through pain, sleep disruption and inflammatory signaling, but the dose–response relationships and mechanistic pathways by which facial injuries modify pbTBI remain poorly defined [174,175,176].

By contrast, the neurocranium encloses the brain in a rigid container and introduces layered membranes and fluids (i.e., dura, arachnoid, subarachnoid CSF, and pia) that create repeated impedance boundaries and physiological barriers [172]. The meningeal complex is not only structural but also immunological, and its integrity shapes how immune signals and cells access the CNS [177,178]. Interfaces between the skull, dura, arachnoid barrier, subarachnoid CSF, pia, and the glia limitans combine marked impedance differences with barrier functions that regulate molecular trafficking and immune surveillance [179,180,181]. Finite element and fluid dynamics models suggest that energy dissipation can be concentrated at these interfaces, and at higher intensities skull flexure may transmit pressure waves into CSF and blood compartments, thereby driving secondary intracranial gradients [94,149,182]. Although the blast intensity required to produce meaningful skull deformation remains uncertain [149,182], even small perturbations of meningeal barriers could alter immune cell trafficking and local cytokine milieus in ways that amplify subsequent inflammatory cascades [183,184,185].

The cerebrum is highly heterogeneous in both cytoarchitecture and vascular organization, and this heterogeneity likely contributes to regional vulnerability patterns after blast exposure [186,187,188]. Structural and functional neuroimaging studies report region-specific alterations after blast-related mild TBI, including changes in cortical thickness and disruptions of resting-state functional organization across prefrontal, frontal, parietal, and occipital regions, as well as involvement of the insula, cingulate, hippocampus, and thalamus [189,190,191]. A common mechanistic observation is that many implicated cortical regions are close to the skull and meningeal complex, whereas subcortical regions, such as thalamus and hippocampus, are adjacent to ventricular CSF spaces, creating distinct sets of impedance boundaries at which energy could be localized [149,172]. Rodent studies support time-dependent and exposure-count-dependent microstructural changes, with certain regions, such as the thalamus, showing evidence of cumulative effects after repeated mild exposures [192,193,194]. Recent multimodal imaging has further implicated the rostral anterior cingulate cortex as susceptible to repeated blast, potentially because of its anatomical location near the orbits, and has linked cumulative exposure to neurocognitive outcomes and neuroinflammatory signals measured with TSPO ligands [195,196].

The cerebellum has emerged as a particularly vulnerable structure in blast-related injury, and its involvement provides a useful test case for interface-driven hypotheses because of its posterior location and intimate proximity to the occipital bone [197,198,199,200]. Across clinical imaging studies, reproducible abnormalities have been reported in cerebellar white matter, including structural changes and atrophy in cerebellar peduncles that serve as major conduits between the cerebellum, brainstem, and cerebrum [191,197,201]. These structural findings are complemented by metabolic evidence, including FDG-PET reports of reduced glucose metabolism in cerebellum and related brainstem regions in blast-exposed veterans, with some studies reporting exposure-count correlations [66,67,202]. Preclinical models indicate that repeated exposures may be required to trigger robust cerebellar pathology and that BBB microlesions, Purkinje cell injury, tau-related changes, and persistent gliosis can evolve over weeks, consistent with delayed cell death and neuroimmune processes [66,164,203,204]. Functionally, cerebellar involvement can manifest as subtle deficits in ocular motor processes, motor learning, and sensorimotor gating, as well as deficits in cognitive processing speed, informational timing, attention, and working memory, consistent with cerebellar cognitive affective syndrome [205,206,207,208,209]. Together, these observations reinforce the clinical relevance of a region often underappreciated in blast injury frameworks.

Endocrine structures, particularly the pituitary, also merit consideration because of their anatomic confinement within bone at the skull base and their sensitivity to vascular and mechanical perturbation [210,211,212,213,214]. Post-traumatic hypopituitarism has been reported after blast-related TBI, with abnormalities in growth hormone and IGF-I axes, among others, and animal models support that repeated mild blasts can also perturb hormonal regulation [212,213,215]. Pituitary vulnerability is therefore consistent with the concept that confined spaces and adjacent bony boundaries can create local impedance conditions that favor energy concentration and microvascular disruption [21,210,216]. Endocrine dysfunction can then contribute to impaired recovery by affecting sleep quality, mood, metabolic homeostasis, and vascular reactivity, potentially amplifying PCS-like trajectories [217,218,219].

Intracranial fluid compartments provide another set of high-contrast interfaces that can couple strongly to primary blast, including the CSF in the subarachnoid space and ventricles and blood within the cerebral circulation [220,221,222]. The subarachnoid space, with its trabecular architecture and dynamic fluid movement, cushions the brain under conventional mechanical loads, yet models suggest that it can also serve as a locus for blast energy dissipation and pressure-gradient formation [94,172,223]. Ependymal surfaces lining the ventricles and the subventricular zone represent biologically active interfaces where blast-induced perturbations can alter gene expression programs, including endothelial-related markers such as PECAM1/CD31 and von Willebrand factor (vWF), consistent with vascular and barrier signaling engagement [224,225,226]. Large-animal studies provide striking support for periventricular vulnerability, reporting axonal injury markers concentrated around ventricular walls even when high-field MRI fails to detect structural lesions, and showing that repeated exposures increase both axonal and astroglial pathologies in this region [133,227].

The choroid plexus and blood–CSF barrier represent specialized vascular interfaces that are plausibly vulnerable to blast-wave perturbation and that can influence downstream neuroimmune and homeostatic dynamics [228,229,230]. Experimental studies have reported widened junctions between barrier cells and increased numbers and altered morphology of epiplexus macrophage-like cells after blast exposure, suggesting barrier compromise and immune activation [231,232,233]. Because the blood–CSF barrier regulates the molecular composition of CSF, its disruption can permit entry or retention of inflammatory mediators and cells that alter periventricular environments, increasing risk of neurodegeneration [234,235]. These barrier dynamics connect naturally to the clinical emphasis on temporal evolution, because early barrier perturbations may resolve while leaving behind altered immune set points that shape delayed responses [236,237,238,239]. They also provide a mechanistic rationale for comparing systemic blood biomarkers with CSF profiles, where discordant signatures could help separate predominantly peripheral inflammation from neuroinflammation driven by barrier-localized processes [84,240,241].

Among all intracranial interfaces, the cerebral vasculature is the largest distributed boundary between biofluids and brain tissue, and it therefore represents a compelling primary biological substrate for pbTBI [242,243,244]. The vascular tree spans large arteries and veins, pial arterioles with perivascular spaces, penetrating vessels, and an enormous capillary network whose total luminal surface area is estimated at tens of square meters [245,246,247]. Across this tree, cellular composition and gene expression are spatially heterogeneous, with endothelial phenotypes varying by vessel class and with additional complexity introduced by smooth muscle layers, pericyte and astrocyte coverage, and perivascular macrophage populations [188,248,249]. This diversity matters because blast-wave energy deposition at blood-vessel wall interfaces could produce regional and vessel-class-specific patterns of endothelial vulnerability, regional dysperfusion, and immune signaling [250,251,252,253].

The neurovascular unit (NVU) comprises capillary endothelial cells, pericytes, basement membranes, astrocytic endfeet, neurons, and microglia organized to support BBB integrity, metabolic coupling, and activity-dependent regulation of blood flow [254]. BBB integrity depends on tight junction proteins such as claudin-5 and occludin, scaffolding proteins including ZO-1/ZO-2, and adherens junction components such as VE-cadherin, all of which maintain a high-resistance barrier against paracellular flux [255,256,257]. Multiple studies report increased circulating levels of junction proteins after mild blast exposures, consistent with junctional disruption and suggesting that barrier perturbation can occur even when parenchymal imaging is normal [75,258,259]. Pericytes add another layer of vulnerability because they are mechanically integrated with endothelial basement membranes via unique junctional structures and because they regulate capillary tone, barrier stability, angiogenesis, and immune signaling in a vessel-class-dependent manner [260,261,262]. Ultrastructural studies in animal models describe time-dependent changes across NVU elements, including altered luminal area, endothelial and astrocytic morphology changes, and pericyte degeneration signatures, which collectively support a dynamic and indeterminant injury–repair continuum rather than a static lesion model [228,263,264].

Perivascular spaces and glymphatic system function depend on vascular pulsatility to remove brain waste and maintain sleep-dependent homeostasis [265,266,267]. Perivascular spaces are fluid-filled compartments surrounding arterioles and venules that interface the CSF with interstitial fluid, and their microfluidic dynamics depend on cardiac-driven vessel pulsations and intact astrocytic aquaporin-4 (AQP4) polarization at endfeet [265,266,268]. Enlarged perivascular spaces on MRI are interpreted as markers of glymphatic dysfunction and have been reported in blast-exposed cohorts, sometimes in association with neuropsychological impairment and sleep disruption [269,270,271]. Experimental work and postmortem analyses suggest that blast exposure can alter AQP4 expression and, critically, its perivascular localization, thereby impairing clearance even when total protein levels are not dramatically changed [271,272,273]. The functional significance of AQP4 mislocalization in human pbTBI remains inferred rather than demonstrated; MRI perivascular space enlargement is an indirect proxy measure with multiple non-blast etiologies, and direct measurement of glymphatic clearance rates in blast-exposed humans is not yet feasible with current methods.

At the subcellular level, axons and synapses represent additional interfaces where rapid mechanical perturbations could translate into functional disruption, and these structures can be engaged both directly by pressure dynamics and indirectly through vascular and immune cascades [252,274,275]. Axonal injury markers have been detected in primary blast models, with some large-animal work showing periventricular white matter injury and cumulative effects with repeated exposures, consistent with interface-driven biomechanics at ventricular and vascular boundaries [133,276,277]. Secondary mechanisms, including neuroinflammation, oxidative stress, and excitotoxicity, can then amplify initial axonal transport disruption and contribute to prolonged degeneration, linking acute exposure to delayed dysfunction trajectories [278,279,280]. Synapses are specialized cell–cell junctions stabilized by adhesion molecules and cytoskeletal partners, and modeling suggests that millisecond-scale pressure waves could transiently misalign pre- and postsynaptic elements, thereby disrupting receptor localization and neurotransmission [281]. Rodent and slice-culture studies report dose- and time-dependent alterations in synaptic proteins and ultrastructure after blast exposure, supporting the concept that nanoscale perturbations can coexist with primarily vascular injury signatures and jointly shape cognitive outcomes [141,282,283].

Identifying the most vulnerable biological substrates of pbTBI is complicated by methodological asymmetries and by the inherently time-dependent nature of blast-induced changes. Clinical imaging studies often emphasize macroscopic anatomy and network-level function, whereas experimental studies frequently focus on localized histology and ultrastructure, making it difficult to map findings across scales and species [284,285,286]. Human studies are typically cross-sectional snapshots at heterogeneous post-exposure intervals, and exposure reconstruction is often incomplete, both of which obscure temporal trajectories and inflate between-study variability [20,287,288]. Animal models remain indispensable but face limitations in anatomy, physiology, and time-scaling, and shock-tube exposures may not replicate the waveform complexity of open-field or confined-space blasts experienced in operational settings [167,289]. The biological substrates and their vulnerability patterns are summarized in Table 1 (biological substrates rows).

## 7. The Pathobiology and Pathophysiology of pbTBI

**Figure 3:**
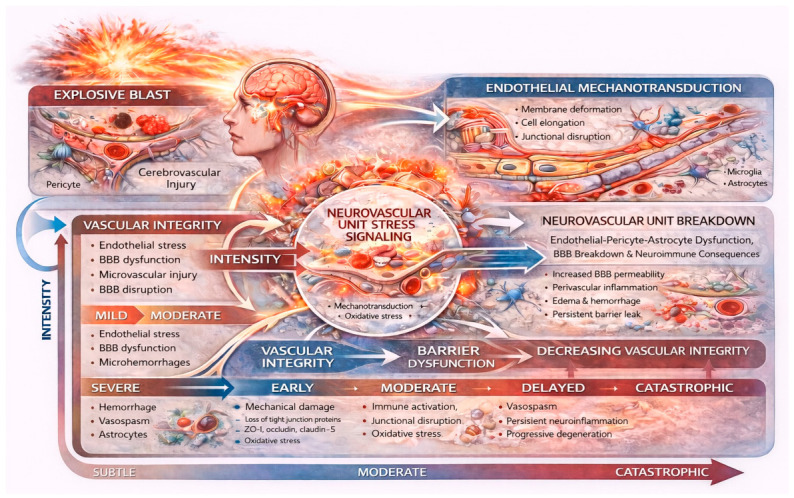
Neurovascular mechanisms underlying primary blast-related traumatic brain injury (pbTBI). Conceptual schematic illustrating how explosive blast overpressure induces cerebrovascular and neurovascular unit (NVU) injury. Mechanical forces trigger endothelial mechanotransduction, leading to membrane deformation, cellular elongation, and junctional disruption. NVU stress signaling involves endothelial cells, pericytes, astrocytes, and immune cells, producing oxidative stress and neuroinflammation. Blast intensity determines progression from early endothelial stress and blood–brain barrier (BBB) dysfunction to full NVU breakdown, with increased BBB permeability, perivascular inflammation, edema, hemorrhage, and persistent barrier leakage. The lower panel reflects temporal progression from early mechanical injury to delayed vasospasm, chronic neuroinflammation, and progressive neurodegeneration. The figures were created using ChatGPT Version 1.2026.048 (1771630681).

We advance the hypothesis that cerebrovascular injury and endothelial stress constitute the dominant early pathobiological response to primary blast. This hypothesis is supported by converging evidence from human biomarker studies, postmortem neuropathology, and preclinical models, but it remains incompletely validated and should be weighed against alternative mechanistic frameworks. The pathobiology of pbTBI can be largely interpreted through the lens of cerebrovascular injury and endothelial stress, which align directly with impedance-driven biomechanics and with clinical features across severity levels (Figure 3) [21,228,290]. Cerebrovascular injury in this context encompasses disruptions spanning arteries, arterioles, capillaries, venules, and the NVU–BBB interface, with downstream consequences for perfusion regulation, barrier function, and neuroimmune signaling [164,228,291]. The cerebral vasculature is uniquely specialized relative to peripheral vessels because BBB integrity depends on tight junctions, extensive pericyte coverage and astrocytic endfeet organization that together create a mechanically integrated barrier organ which coordinates parenchymal fluid flow with vascular dynamics [243,262,292]. As a result, even subtle mechanical perturbations can propagate across endothelial, pericytic, and astrocytic elements, contributing to dysfunction that is often disproportionate to the apparent magnitude of external injury [293,294]. While the cerebrovascular framework provides the most parsimonious account of available evidence, it is important to situate it explicitly within a competitive mechanistic landscape. White-matter axonal injury represents a potentially primary, vascular-independent mechanism: human DTI cohort studies consistently document microstructural changes in blast-exposed populations, and experimental models demonstrate axonal pathology in the absence of overt BBB disruption. Microglial neuroinflammation may operate as a potentially autonomous driver, with low-level blast models reporting reactive microgliosis and cytokine release under conditions where clear BBB evidence is absent. The possibility also exists that the vascular hypothesis is most applicable to moderate-to-severe pbTBI, and that neuroinflammatory and synaptic mechanisms may predominate at the subconcussive end of the exposure spectrum where gross deformation is absent and endothelial perturbation may be subtle (Box 7). Acknowledging these alternative mechanisms does not weaken the cerebrovascular framework; rather, it reflects the mechanistic plurality that characterizes a heterogeneous exposure spectrum and the likelihood that dominant pathobiological axes shift with injury severity.

Box 7 **Clinical pathophysiology (phenotypes and trajectories).**

Clinical expression of pbTBI is often delayed, variable, and shaped by both exposure dosing and host vulnerability.Severity spectrum: from severe blast injury with acute edema/hyperemia and delayed vasospasm to mild/subconcussive exposures with minimal immediate symptoms.Repeated low-level blasts can produce cumulative risk with heterogeneous delayed outcomes (cognitive, affective, sleep, vestibular, and headache).Persistent symptom trajectories (PCS/PPCS) reflect interacting drivers: vascular dysregulation, neuroinflammation, network dysfunction, and comorbidities.Clinical considerations: integrate exposure history (magnitude, frequency, and setting) with multimodal biomarkers (vascular physiology, diffusion microstructure, and functional connectivity).

Dependencies between blast intensities and vascular outcomes can produce qualitatively different biological outputs as intensity increases. At high intensities, direct mechanical disruption can rupture vessels, destroy endothelial membranes, detach pericytes, and compromise smooth muscle function, producing hemorrhage, hyperemia, edema, and ischemic-hemorrhagic patterns that resemble vascular catastrophes [21,295,296]. Survivors of severe blast exposure may then transition into delayed phases characterized by vasospasm, chronic neuroinflammation, and progressive structural and functional changes, consistent with a cascade model in which early vascular injury seeds later degeneration [34,264,290]. At moderate intensities, microvascular injury, BBB disruption, and microhemorrhages can occur without gross edema, yielding a phenotype that is clinically significant but mechanistically more challenging to detect with conventional imaging [164,232,297]. At mild and subconcussive intensities, endothelial stress and junctional perturbation may be the dominant early events, and their clinical relevance emerges through cumulative exposures and delayed or state-dependent symptom expression rather than immediate catastrophic deficits [164,239,252,253,298].

Endothelial cells integrate mechanical forces into biochemical and transcriptional responses that can destabilize the NVU even when structural damage is subtle [299,300,301]. (Figure 3, upper panel). Blast overpressure can compress endothelial membranes, deform basement membranes, and perturb intercellular junctions, producing immediate barrier dysfunction that may precede or occur independently of inflammation. Abnormal shear patterns and pulsatility changes can further activate mechanotransduction pathways that reorganize the cytoskeleton, alter junction protein localization, and modulate transcellular transport pathways [302,303,304]. Because the NVU couples endothelial, pericytic, and astrocytic signaling, endothelial stress can disrupt neurovascular and gliovascular coupling, impair activity-dependent blood flow regulation, and disturb glioneuronal metabolic support [305,306,307].

BBB dysfunction after blast exposure is frequently conceptualized as biphasic, with an early phase driven by mechanical disruption and a delayed phase driven by inflammatory and oxidative processes [164,308] (Figure 2). These temporal phases are severity-dependent, and divergence between mild and severe trajectories becomes most pronounced at two to four weeks post-exposure, as illustrated in Figure 2. Early changes include loss or mislocalization of junction proteins, such as claudin-5, occludin, ZO-1, and VE-cadherin, which increases paracellular permeability and can promote vasogenic edema even when imaging appears normal [152,309]. Delayed changes can involve junctional disruption and destabilization [152,164,310], altered efflux and receptor-mediated transport [311,312], basement membrane changes, and persistent perivascular immune activation [252,264,290], which together sustain barrier leak and impair homeostatic trafficking. Importantly, barrier disruption need not be binary; subtle increases in permeability can meaningfully alter perivascular cytokine gradients, microglial activation thresholds, and glymphatic clearance efficiency [272,313,314].

Endothelial stress also perturbs hemostatic balance and vascular tone, linking blast exposure to thrombo-inflammatory states that can amplify microvascular dysfunction [315,316,317]. Mechanistically, increased vWF release [226], tissue factor upregulation [318], altered thrombomodulin-protein C signaling [319], and elevated PAI-1 [320] can shift the system toward a pro-thrombotic phenotype, while microvascular constriction or dysregulated vasodilation can further compromise perfusion [250,321]. Redox imbalance is a complementary axis, because increased NADPH oxidase activity, reduced antioxidant capacity, and reduced nitric oxide bioavailability can promote vasoconstriction, endothelial activation, and barrier dysfunction [317,322,323]. Metabolic reprogramming toward glycolysis, mitochondrial dysfunction, and altered lipid utilization can then create energy deficits that increase susceptibility to apoptosis or dysfunctional repair, particularly under repeated exposure conditions [324,325,326].

Vascular injury and endothelial stress initiate inflammatory cascades through canonical pathways, such as NF-κB activation, which increases the transcription of cytokines, chemokines, and adhesion molecules that promote leukocyte recruitment [327,328,329]. Endothelial upregulation of ICAM-1, VCAM-1, and selectins, together with elevated chemokines, such as MCP-1, can promote vascular wall inflammation and, when barrier integrity is compromised, facilitate the infiltration of peripheral immune cells into perivascular and parenchymal compartments [312,328,330,331]. Complement activation can be engaged by release of damage-associated molecular patterns, such as HMGB1, in more severe injuries and by downregulation of complement regulatory proteins that normally constrain activation at endothelial surfaces [332,333,334]. In parallel, resident microglia and astrocytes shift toward inflammatory phenotypes, releasing cytokines, chemokines, and reactive oxygen species that amplify injury and can persist beyond resolution of systemic signals [335,336,337]. Because these inflammatory processes are layered over dynamic perfusion and clearance changes, they can produce heterogeneous symptom trajectories that depend on injury intensity, prior exposure history, sleep, and other physiological modifiers [272,338,339].

Distinguishing systemic inflammation from neuroinflammation is clinically important, but the distinction can blur over time as barrier status and immune trafficking evolve (Figure 3, lower panel). In mild pbTBI where the BBB is largely intact, neuroinflammation dominated by microglia and astrocytes may predominate, and symptoms may present as cognitive inefficiency, fatigue, sleep disturbance, and mood dysregulation without systemic inflammatory signs [294,335,340]. In more severe cases of pbTBI with barrier compromise and parenchymal damage, peripheral leukocytes can enter the CNS and interact with resident glia, creating a mixed inflammatory milieu that can include fever and multisystem consequences in addition to neurological deficits [238,331,341]. These compartmental differences imply that biomarker strategies should compare systemic blood and CSF profiles and that treatment windows may differ for peripheral immune modulation versus central glial targeting [342,343,344]. Time-resolved monitoring is therefore essential because early phases may be amenable to barrier-stabilizing and anti-inflammatory interventions, whereas chronic neuroinflammation and glymphatic dysfunction may require longer-term modulation of vascular and clearance physiology [272,337]. A critical gap is determining whether early inflammation impairs or promotes long-term barrier and glymphatic function.

Current evidence implicating vascular injury and endothelial stress in pbTBI is strong but remains limited by translational asymmetries, confounding animal and human data.

Most mechanistic datasets derive from rodent models and shock-tube exposures that may not fully replicate the waveform complexity and whole-body context of real-world blast, and human data are often concentrated at the extremes of severity or in cohorts with heterogeneous co-exposures [12,167,345]. Clinical and experimental outcome measures are frequently mismatched, with laboratory studies emphasizing molecular and ultrastructural endpoints and clinical studies emphasizing symptoms and imaging, which complicates causal inference and triangulation [346,347,348]. Biological modifiers such as age, cardiometabolic risk, prior head trauma, and genetic polymorphisms can reshape inflammatory and vascular responses, yet these are rarely modeled systematically in preclinical work and are often incompletely measured in human cohorts [349,350,351].

**Validity of the Cerebrovascular Framework Under Current Translational Con-straints.** The cerebrovascular framework is best understood as a well-motivated, internally consistent interpretive synthesis rather than a proven mechanism. Each category of supporting evidence carries important limitations. Human biomarker studies are predominantly cross-sectional with heterogeneous exposure reconstructions and rarely isolate primary blast from combined mechanisms; they establish association but not causation. Postmortem neuropathology is concentrated in severe or fatal cases and may not represent the mild-to-subconcussive range most relevant to occupational health. Animal models use lissencephalic rodent brains in shock-tube geometries that differ substantially from open-field or vehicular blasts, with no validated inter-species scaling laws for blast biomechanics. Additionally, computational models require boundary conditions derived from the same experimental systems whose translational validity is under question. Taken together, these limitations mean that the cerebrovascular framework’s primary value is heuristic: it organizes disparate findings, generates testable predictions, and identifies tractable therapeutic targets. Whether it accurately describes the dominant injury axis in mild subconcussive exposures in humans remains an open empirical question.

## 8. Summary of the State of the Science and Important Known Unknowns

Taken together, the state of the science supports the view that pbTBI is a distinct neurotrauma entity with direct relevance to military occupational health. Clinical experience and cohort studies indicate that mild pbTBI and repeated low-level exposures are common, that symptoms are often transient, and that some subjects progress to PCS or PPCS with significant functional impairment [352,353,354]. What remains uncertain is how to define injury thresholds and safe exposure limits when exposures vary across waveform shape, spectral content, and environment, and when individual vulnerability differs by age, prior injury, and physiological reserve. Equally unresolved is the extent and presentation of cumulative risk from repeated low-level exposures, including the interval between exposures, whether exposure counts, impulse-weighted metrics, or physiological response trajectories best predict outcome. Key for operational policy, the field lacks evidence-based guidance on safe inter-exposure intervals and how those intervals change across individuals with increasing prior blast exposure or injury, because interval data are rarely recorded and because the duration of blast-induced cerebral vulnerability has not been established.

From a physics perspective, explosive blast is a multi-component, four-dimensional injurious environment in which peak overpressure is only one descriptor and temporal dynamics determine how energy couples into tissues [17,112,355]. Key unknowns include whether peak blast overpressure is the dominant determinant of injury across the entire pbTBI spectrum or whether other waveform features, such as impulse, rise time, negative phase characteristics, and spectral composition, drive critical aspects of biological injury risk. The relevant biological scale also remains poorly characterized. Current observations of blast effects on systems-level phenomena, such as autoregulation and barrier compliance, have led to assumptions about injury mechanisms at the physiological scale, without full understanding of long-term blast-wave effects at the subcellular level beyond mitochondria in affected cells, especially since the nucleus, endoplasmic reticulum, and Golgi apparatus constitute similar nanoscale acoustic impedance barriers that lack characterization after blast [325,356]. Related to this is the question of which metric, or combination of metrics, best represents a biologically meaningful dose across scales. For the purpose of this review, which doses are relevant for cerebrovascular and NVU injury is critical because current thresholds were often derived from non-neurological endpoints. The field also lacks standardized approaches for measuring and reporting these metrics in realistic environments where reflections, confinement, helmets, and body armor reshape local pressure fields. Finally, reconstructing the injurious environment retrospectively remains difficult, which limits causal inference in human datasets and complicates translation of laboratory waveforms to operational settings. Progress on these questions would facilitate meaningful dose–response biology, but it will only be actionable if it is paired with biomechanical models that specify how particular waveform features map onto intracranial loading patterns.

At the biomechanics and biological substrate levels, the central unknown is which intracranial mechanisms dominate at different intensity ranges and under what tissue microenvironments. It remains unclear whether specific biomechanical responses (e.g., spalling, cavitation, and strain propagation) have identifiable thresholds or whether their contributions vary with waveform intensity. A related question is whether different anatomical structures effectively “filter” or transform the incoming waveform, such that deep structures experience a different frequency and impulse profile than cortical surfaces, and whether this transformation explains regional vulnerability patterns. The relative contribution of direct cranial transmission versus thoracic or vascular mechanisms also remains unresolved, and it may be intensity-dependent, with peripheral mechanisms becoming more relevant as whole-body loading increases. Directionality is an additional under-characterized variable because lateralized exposure can create asymmetric intracranial gradients that may influence unilateral imaging findings and symptom profiles [357,358,359]. Resolving these issues will require in vivo validation tools, including imaging, biosensors, and computational models. Constrained by physiological data, these tools can map blast waveform properties to measured vascular and barrier responses at clinically relevant time scales.

From a translational perspective, the field’s most urgent unknowns concern biomarkers, inflection points, and treatment windows. Identifying biochemical or protein biomarkers that are specific, or at least enriched, for pbTBI relative to other mild TBI mechanisms is critical, particularly biomarkers reflecting endothelial stress, BBB perturbation, and thrombo-inflammation. However, without longitudinal sampling, it remains difficult to determine when biomarker trajectories diverge toward recovery versus worsening and how these inflection points depend on injury severity, exposure density, or physical conditioning. Equally important is the need for deployable diagnostic approaches that can inform return-to-duty decisions and exposure mitigation because the operational cost of missing a vulnerability window may be higher than the cost of short-term restriction. Mechanism-based therapeutics, such as those that target endothelial stabilization, barrier repair, redox modulation, and targeted neuroimmune intervention, are plausible, but their success will depend on identifying optimal treatment windows during recovery.

## 9. Conclusions and Future Directions

Primary blast-induced TBI is biomechanically and biologically distinct from impact TBI because its initiating insult is a pressure waveform that interacts with distributed intracranial fluids and impedance boundaries on millisecond timescales (Table 1). The abrupt pressure transitions, negative phase dynamics, and frequency-dependent energy distribution of blast waves create loading environments that cannot be fully captured by acceleration-based frameworks derived from blunt impact. Across experimental models, neuropathology, and clinical neuroimaging, the cerebrovascular system, especially at the level of the neurovascular unit, functions as a critical energy-absorption interface and is therefore a primary injury substrate. This vascular vulnerability offers a parsimonious explanation for why symptoms can be diffuse and heterogeneous, why conventional imaging can be normal, and why delayed phases such as vasospasm or persistent neuroinflammation can emerge after an apparently minor initial event. It also offers a concrete translational advantage in that vascular and barrier physiology is measurable, modifiable, and already the focus of therapeutic strategies in other neurological diseases, making it an attractive anchor for pbTBI prevention and treatment, especially for chronic subconcussive exposures during weapons system use.

A central conclusion of this review is that vascular injury and endothelial stress constitute a core pathobiological response to primary blast, with downstream consequences that include tight-junction disruption, BBB leak, altered perfusion and pulsatility, impaired neurovascular coupling, and disturbed glymphatic clearance. These vascular perturbations can then initiate innate immune activation, microglial and astrocytic reactivity, and metabolic dysfunction that evolve over days to months and drive key PCS/PPCS trajectories. Under repeated and “subconcussive” exposure patterns typical of weapons system use, these cascades appear to exhibit cumulative and density-dependent behavior, implying that recovery kinetics and exposure spacing are key determinants of long-term risk. Importantly, the vascular model does not exclude neuronal or synaptic injury; rather, it places vascular and perivascular dysfunction upstream in a causal chain that compromises synaptic signaling, axonal transport, and network-level function.

Future progress will depend on redefining how “dose” is measured (and reported) and linked to biology, because peak overpressure alone is insufficient to capture the waveform features most likely to stress vascular and barrier interfaces (Box 8). A practical near-term strategy is to integrate external waveform measures with internal physiological readouts, such as cerebrovascular reactivity, perfusion measures, BBB permeability dynamics, and biomarker trajectories. In parallel, establishing molecular and cellular thresholds for endothelial stress and NVU disruption in blast-naïve versus blast-experienced persons can enable biodosimetry approaches that support individualized risk stratification. Mechanistic validation will require in vivo studies to validate candidate biomechanical mechanisms by linking measured intracranial pressure and vascular responses to subsequent barrier, immune, and behavioral outcomes under operationally relevant exposures.

Box 8 **Minimum Dataset Requirements for Mechanistically Informative pbTBI Studies.**

Exposure characterization (non-negotiable): Waveform recording must include peak overpressure AND impulse AND positive-phase duration as a minimum; environmental descriptors (open-field vs. confined); for repeated-exposure designs, both exposure count and inter-exposure interval must be recorded prospectively.

Biological sampling (non-negotiable): A minimum of three timepoints is required—acute (≤24 h), subacute (72 h–2 weeks), and delayed (1–3 months)—to capture both early mechanical and delayed inflammatory phases. Paired peripheral blood and CSF sampling is preferred. Biomarker panels must include CNS-specific markers (GFAP, NfL, and UCH-L1) AND vascular/endothelial markers (VE-cadherin fragments, VEGF, angiopoietin-2, von Willebrand factor, etc.) to enable mechanistic attribution.

Human study requirements (non-negotiable): A pre-exposure baseline is mandatory for repeated-exposure designs. PTSD screening with a validated instrument is required for covariate modeling. Sex must be reported as a biological variable with powered stratification or explicit acknowledgment of the limitation. Audiological assessment (tympanometry plus pure-tone audiogram) should be included as a blast-specific screen. Studies that do not meet these minimum standards should be interpreted as hypothesis-generating rather than mechanistically definitive.

Biomarker and therapeutic development is expected to accelerate, with a focus on vascular and NVU biology, emphasizing easily accessible and robust markers of endothelial activation, junctional disruption, thrombo-inflammation, and glymphatic dysfunction, complemented by perfusion and permeability imaging when possible. Mechanism-based therapeutics aimed at stabilizing endothelial function, repairing BBB integrity, modulating neuroinflammation, and correcting metabolic dysfunction are high-priority avenues, particularly because many cascades unfold over days and therefore may present actionable therapeutic windows. Longitudinal clinical and preclinical studies are essential to determine whether repeated blast exposure contributes to neurodegenerative trajectories, endocrine dysfunction, and progressive cognitive decline and to establish whether these outcomes are mediated by persistent vascular dysfunction and impaired glymphatic clearance. To support such work, experimental models must better replicate real-world blast physics, including overpressure and repetition ranges, environmental reflections, frequency content, and whole-body exposure, so that mechanistic insights translate to operationally relevant recommendations.

## Figures and Tables

**Table 1:** Summary of biomechanics. Evidentiary tier, human data availability, operative intensity range, and preventability of the four candidate biomechanical mechanisms of primary blast-induced traumatic brain injury.

Mechanism	Primary Evidence Base	Human Data?	Intensity Range	Preventable?
Shear	DTI cohorts; kinetic-TBI analogy	Yes—multiple human cohorts	Moderate–high	Partial (helmet design)
Spalling	Postmortem neuropathology; impedance physics	Indirect (autopsy only)	High	Not currently feasible
Cavitation	Computational models; ex vivo preparations	Not validated in vivo	Low–moderate	Very difficult
Nanoscale strain	Theoretical modeling only	None	Subconcussive	Unknown

**Table 2:** Multiscale framework of primary blast–related traumatic brain injury (pbTBI): clinical manifestations, physical blast characteristics, biomechanical mechanisms, biological substrates, and pathobiological processes.

Clinical	Physics	Biomechanics	Biological Substrates	Pathobiology
**Symptomatology:**	**Components:**	**Mechanisms:**	**Structures:**	**Mechanisms**
**Severe:** GCS 3–8, prolonged unconsciousness up to 3 weeks and coma and post-traumatic amnesia, severe lasting neurological impairment **Moderate:** GCS 9–12, altered consciousness up to 24 h, post-traumatic amnesia up to 7 days, alteration of daily function and deficits in concentration deficits, impaired executive functions **Mild and “sub-concussive”:** *GCS* 14–15, altered consciousness (<30 min), acute and transient headache, dizziness, nausea, confusion, impaired memory, balance, concentration, vision, tinnitus, irritability, PCS and PPCS	**Peak overpressure;** (in PSI or kPa) **Impulse;** total energy delivered over time **Duration;** length of the pressure wave **Frequency;** spectral components (Hz–kHz)	**Spalling** **Shear stress;** **Cavitation** **Implosion;** **Nanoscale tissue injury**	Peripheral: Thorax Abdomen Central: Head Viscerocranium Neurocranium Cerebrum Cerebellum Pituitary Cerebral ventricles Cerebral vasculature Perivascular space Microanatomical Cell adhesion Neurovascular unit Chemical synapse	Cerebrovascular injury; Endothelial stress; Inflammation (Oxidative stress; Mitochondrial dysfunction; Axonal injury)

## Data Availability

No new data were created or analyzed in this study. The authors had no role in the design of the referenced studies; in the collection, analyses, or interpretation of data; in the writing of the manuscript; or in the decision to publish the results except their own. The views, information, or content and conclusions presented do not necessarily represent the official position, or policy of, nor should any official endorsement be inferred on the part of, the Uniformed Services University, the Department of War (previously named the Department of Defense), the US Department of Veterans Affairs, the United States Government, or any affiliated agencies.

## References

[1] Ommaya A.K., Ommaya A.K., Dannenberg A.L., Salazar A.M. (1996). Causation, incidence, and costs of traumatic brain injury in the U.S. military medical system. J. Trauma Acute Care Surg..

[2] Lindberg M.A., Moy Martin E.M., Marion D.W. (2022). Military Traumatic Brain Injury: The History, Impact, and Future. J. Neurotrauma.

[3] Kinch K., Fullerton J.L., Stewart W. (2019). One hundred years (and counting) of blast-associated traumatic brain injury. J. R. Army Med. Corps.

[4] French L.M., Taber K.H., Helmick K., Hurley R.A., Warden D.L. (2015). Traumatic Brain Injury in the Military Population. Combat and Operational Behavioral Health.

[5] DePalma R.G., Kobeissy F.H. (2015). Combat TBI: History, Epidemiology, and Injury Modes. Brain Neurotrauma: Molecular, Neuropsychological, and Rehabilitation Aspects.

[6] Defense and Veterans Brain Injury Center, (DVBIC). DoD Worldwide Numbers for TBI. https://health.mil/Military-Health-Topics/Centers-of-Excellence/Traumatic-Brain-Injury-Center-of-Excellence/DOD-TBI-Worldwide-Numbers.

[7] Lindquist L.K., Love H.C., Elbogen E.B. (2017). Traumatic Brain Injury in Iraq and Afghanistan Veterans: New Results from a National Random Sample Study. J. Neuropsychiatry Clin. Neurosci..

[8] Vickers M.L., Coorey C.P., Milinovich G.J., Eriksson L., Assoum M., Reade M.C. (2018). Bibliometric analysis of military trauma publications: 2000–2016. J. R. Army Med. Corps.

[9] Bass C.R., Panzer M.B., Rafaels K.A., Wood G., Shridharani J., Capehart B. (2012). Brain injuries from blast. Ann. Biomed. Eng..

[10] Cernak I., Noble-Haeusslein L.J. (2010). Traumatic brain injury: An overview of pathobiology with emphasis on military populations. J. Cereb. Blood Flow Metab..

[11] DePalma R.G., Hoffman S.W. (2018). Combat blast related traumatic brain injury (TBI): Decade of recognition; promise of progress. Behav. Brain Res..

[12] Bryden D.W., Tilghman J.I., Hinds S.R. (2019). Blast-Related Traumatic Brain Injury: Current Concepts and Research Considerations. J. Exp. Neurosci..

[13] Siedhoff H.R., Chen S., Song H., Cui J., Cernak I., Cifu D.X., DePalma R.G., Gu Z. (2021). Perspectives on Primary Blast Injury of the Brain: Translational Insights Into Non-inertial Low-Intensity Blast Injury. Front. Neurol..

[14] Agoston D., Arun P., Bellgowan P., Broglio S., Cantu R., Cook D., da Silva U.O., Dickstein D., Elder G., Fudge E. (2017). Military Blast Injury and Chronic Neurodegeneration: Research Presentations from the 2015 International State-of-the-Science Meeting. J. Neurotrauma.

[15] Cernak I., Savic J., Ignjatovic D., Jevtic M. (1999). Blast injury from explosive munitions. J. Trauma Acute Care Surg..

[16] Masel B.E., Bell R.S., Brossart S., Grill R.J., Hayes R.L., Levin H.S., Rasband M.N., Ritzel D.V., Wade C.E., DeWitt D.S. (2012). Galveston Brain Injury Conference 2010: Clinical and experimental aspects of blast injury. J. Neurotrauma.

[17] Cernak I. (2017). Understanding blast-induced neurotrauma: How far have we come?. Concussion.

[18] Agoston D.V., Elsayed M. (2012). Serum-Based Protein Biomarkers in Blast-Induced Traumatic Brain Injury Spectrum Disorder. Front. Neurol..

[19] DePalma R.G., Burris D.G., Champion H.R., Hodgson M.J. (2005). Blast injuries. N. Engl. J. Med..

[20] Phipps H., Mondello S., Wilson A., Dittmer T., Rohde N.N., Schroeder P.J., Nichols J., McGirt C., Hoffman J., Tanksley K. (2020). Characteristics and Impact of U.S. Military Blast-Related Mild Traumatic Brain Injury: A Systematic Review. Front. Neurol..

[21] Nakagawa A., Manley G.T., Gean A.D., Ohtani K., Armonda R., Tsukamoto A., Yamamoto H., Takayama K., Tominaga T. (2011). Mechanisms of primary blast-induced traumatic brain injury: Insights from shock-wave research. J. Neurotrauma.

[22] Nakagawa A., Ohtani K., Goda K., Kudo D., Arafune T., Washio T., Tominaga T. (2016). Mechanism of Traumatic Brain Injury at Distant Locations After Exposure to Blast Waves: Preliminary Results from Animal and Phantom Experiments. Acta Neurochir. Suppl..

[23] Meaney D.F., Smith D.H. (2011). Biomechanics of concussion. Clin. Sports Med..

[24] Orr T.J., Lesha E., Kramer A.H., Cecia A., Dugan J.E., Schwartz B., Einhaus S.L. (2024). Traumatic Brain Injury: A Comprehensive Review of Biomechanics and Molecular Pathophysiology. World Neurosurg..

[25] Hicks R.R., Fertig S.J., Desrocher R.E., Koroshetz W.J., Pancrazio J.J. (2010). Neurological effects of blast injury. J. Trauma Acute Care Surg..

[26] Eastridge B.J., Mabry R.L., Seguin P., Cantrell J., Tops T., Uribe P., Mallett O., Zubko T., Oetjen-Gerdes L., Rasmussen T.E. (2012). Death on the battlefield (2001–2011): Implications for the future of combat casualty care. J. Trauma Acute Care Surg..

[27] Armonda R.A., Bell R.S., Vo A.H., Ling G., DeGraba T.J., Crandall B., Ecklund J., Campbell W.W. (2006). Wartime traumatic cerebral vasospasm: Recent review of combat casualties. Neurosurgery.

[28] Yu X., Nguyen T.T., Wu T., Ghajari M. (2022). Non-Lethal Blasts can Generate Cavitation in Cerebrospinal Fluid While Severe Helmeted Impacts Cannot: A Novel Mechanism for Blast Brain Injury. Front. Bioeng. Biotechnol..

[29] Lee W.L., Alias A., Lim M.S. (2024). Case Report and Literature Review: A Severe Case of Blast-Related Traumatic Brain Injury. Asian J. Neurosurg..

[30] Colamaria A., Blagia M., Carbone F., Fochi N.P. (2022). Blast-related traumatic brain injury: Report of a severe case and review of the literature. Surg. Neurol. Int..

[31] Martindale S.L., Kolaja C.A., Belding J.N., Liu L., Rull R.P., Trone D.W., Rowland J.A. (2025). Blast exposure and long-term diagnoses among veterans: A millennium cohort study investigation of high-level blast and low-level blast. Front. Neurol..

[32] Rosenfeld J.V., McFarlane A.C., Bragge P., Armonda R.A., Grimes J.B., Ling G.S. (2013). Blast-related traumatic brain injury. Lancet Neurol..

[33] Ling G., Bandak F., Armonda R., Grant G., Ecklund J. (2009). Explosive blast neurotrauma. J. Neurotrauma.

[34] Alford P.W., Dabiri B.E., Goss J.A., Hemphill M.A., Brigham M.D., Parker K.K. (2011). Blast-induced phenotypic switching in cerebral vasospasm. Proc. Natl. Acad. Sci. USA.

[35] Salehi A., Zhang J.H., Obenaus A. (2017). Response of the cerebral vasculature following traumatic brain injury. J. Cereb. Blood Flow Metab..

[36] Biagianti B., Stocchetti N., Brambilla P., Van Vleet T. (2020). Brain dysfunction underlying prolonged post-concussive syndrome: A systematic review. J. Affect. Disord..

[37] Bogdanova Y., Verfaellie M. (2012). Cognitive sequelae of blast-induced traumatic brain injury: Recovery and rehabilitation. Neuropsychol. Rev..

[38] Sayer N.A., Chiros C.E., Sigford B., Scott S., Clothier B., Pickett T., Lew H.L. (2008). Characteristics and rehabilitation outcomes among patients with blast and other injuries sustained during the Global War on Terror. Arch. Phys. Med. Rehabil..

[39] Sayer N.A. (2012). Traumatic brain injury and its neuropsychiatric sequelae in war veterans. Annu. Rev. Med..

[40] Porter K.E., Stein M.B., Martis B., Avallone K.M., McSweeney L.B., Smith E.R., Simon N.M., Gargan S., Liberzon I., Hoge C.W. (2018). Postconcussive symptoms (PCS) following combat-related traumatic brain injury (TBI) in Veterans with posttraumatic stress disorder (PTSD): Influence of TBI, PTSD, and depression on symptoms measured by the Neurobehavioral Symptom Inventory (NSI). J. Psychiatr. Res..

[41] Rickards T.A., Cranston C.C., McWhorter J. (2022). Persistent post-concussive symptoms: A model of predisposing, precipitating, and perpetuating factors. Appl. Neuropsychol. Adult.

[42] Leddy J.J., Haider M.N., Noble J.M., Rieger B., Flanagan S., McPherson J.I., Shubin-Stein K., Saleem G.T., Corsaro L., Willer B. (2021). Clinical Assessment of Concussion and Persistent Post-Concussive Symptoms for Neurologists. Curr. Neurol. Neurosci. Rep..

[43] Permenter C.M., Fernández-de Thomas R.J., Sherman A.L. (2025). Postconcussive Syndrome. StatPearls.

[44] Boake C., McCauley S.R., Levin H.S., Pedroza C., Contant C.F., Song J.X., Brown S.A., Goodman H., Brundage S.I., Diaz-Marchan P.J. (2005). Diagnostic criteria for postconcussional syndrome after mild to moderate traumatic brain injury. J. Neuropsychiatry Clin. Neurosci..

[45] King N. (2014). Permanent post concussion symptoms after mild head injury: A systematic review of age and gender factors. NeuroRehabilitation.

[46] Williams J.L., McDevitt-Murphy M.E., Murphy J.G., Crouse E.M. (2017). Postconcussive Symptoms, PTSD, and Medical Disease Burden in Treatment-Seeking OEF/OIF/OND Veterans. Mil. Med..

[47] Gard A., Vedung F., Piehl F., Khademi M., Wernersson M.P., Rorsman I., Tegner Y., Pessah-Rasmussen H., Ruscher K., Marklund N. (2023). Cerebrospinal fluid levels of neuroinflammatory biomarkers are increased in athletes with persistent post-concussive symptoms following sports-related concussion. J. Neuroinflammation.

[48] Tagge C.A., Fisher A.M., Minaeva O.V., Gaudreau-Balderrama A., Moncaster J.A., Zhang X.L., Wojnarowicz M.W., Casey N., Lu H., Kokiko-Cochran O.N. (2018). Concussion, microvascular injury, and early tauopathy in young athletes after impact head injury and an impact concussion mouse model. Brain.

[49] Jaffee M.S., Meyer K.S. (2009). A brief overview of traumatic brain injury (TBI) and post-traumatic stress disorder (PTSD) within the Department of Defense. Clin. Neuropsychol..

[50] Chen Y., Huang W. (2011). Non-impact, blast-induced mild TBI and PTSD: Concepts and caveats. Brain Inj..

[51] Loignon A., Ouellet M.C., Belleville G. (2020). A Systematic Review and Meta-analysis on PTSD Following TBI Among Military/Veteran and Civilian Populations. J. Head Trauma Rehabil..

[52] Pérez Garcia G., Perez G.M., De Gasperi R., Gama Sosa M.A., Abutarboush R., Kawoos U., Zhu C., Toro C.A., Hof P.R., Ahlers S.T. (2025). Minocycline partially reverses established PTSD-related behavioral traits in rats exposed to repetitive low-level blast injury. Front. Neurol..

[53] Centers for Disease Control and Prevention Comprehensive Listing ICD-10-CM Files. https://www.cdc.gov/nchs/icd/icd-10-cm/files.html.

[54] Tovar M.A., Bell R.S., Neal C.J. (2021). Epidemiology of Blast Neurotrauma: A Meta-analysis of Blast Injury Patterns in the Military and Civilian Populations. World Neurosurg..

[55] Spencer R.W., Brokaw E., Carr W., Chen Z.J., Garfield B.A., Garimella H.T., Gharahi H., Iampaglia J., Lalis L., Przekwas A. (2023). Fiscal Year 2018 National Defense Authorization Act, Section 734, Weapon Systems Line of Inquiry: Overview and Blast Overpressure Tool-A Module for Human Body Blast Wave Exposure for Safer Weapons Training. Mil. Med..

[56] Silverberg N.D., Iaccarino M.A., Panenka W.J., Iverson G.L., McCulloch K.L., Dams-O’Connor K., Reed N., McCrea M. (2020). Management of Concussion and Mild Traumatic Brain Injury: A Synthesis of Practice Guidelines. Arch. Phys. Med. Rehabil..

[57] Eskridge S.L., Macera C.A., Galarneau M.R., Holbrook T.L., Woodruff S.I., Macgregor A.J., Morton D.J., Shaffer R.A. (2013). Combat blast injuries: Injury severity and posttraumatic stress disorder interaction on career outcomes in male servicemembers. J. Rehabil. Res. Dev..

[58] Luethcke C.A., Bryan C.J., Morrow C.E., Isler W.C. (2011). Comparison of concussive symptoms, cognitive performance, and psychological symptoms between acute blast-versus nonblast-induced mild traumatic brain injury. J. Int. Neuropsychol. Soc..

[59] Norris J.N., Sams R., Lundblad P., Frantz E., Harris E. (2014). Blast-related mild traumatic brain injury in the acute phase: Acute stress reactions partially mediate the relationship between loss of consciousness and symptoms. Brain Inj..

[60] Lippa S.M., Pastorek N.J., Benge J.F., Thornton G.M. (2010). Postconcussive symptoms after blast and nonblast-related mild traumatic brain injuries in Afghanistan and Iraq war veterans. J. Int. Neuropsychol. Soc. JINS.

[61] Fausti S.A., Wilmington D.J., Gallun F.J., Myers P.J., Henry J.A. (2009). Auditory and vestibular dysfunction associated with blast-related traumatic brain injury. J. Rehabil. Res. Dev..

[62] Lew H.L., Garvert D.W., Pogoda T.K., Hsu P.T., Devine J.M., White D.K., Myers P.J., Goodrich G.L. (2009). Auditory and visual impairments in patients with blast-related traumatic brain injury: Effect of dual sensory impairment on Functional Independence Measure. J. Rehabil. Res. Dev..

[63] Vagnozzi R., Signoretti S., Cristofori L., Alessandrini F., Floris R., Isgro E., Ria A., Marziali S., Zoccatelli G., Tavazzi B. (2010). Assessment of metabolic brain damage and recovery following mild traumatic brain injury: A multicentre, proton magnetic resonance spectroscopic study in concussed patients. Brain.

[64] Vagnozzi R., Signoretti S., Tavazzi B., Cimatti M., Amorini A.M., Donzelli S., Delfini R., Lazzarino G. (2005). Hypothesis of the postconcussive vulnerable brain: Experimental evidence of its metabolic occurrence. Neurosurgery.

[65] Prins M.P.D., Alexander D., Giza C.C., Hovda D. (2012). Repeat Mild Traumatic Brain Injury: Mechanisms of Cerebral Vulnerability. J. Neurotrauma.

[66] Meabon J.S., Huber B.R., Cross D.J., Richards T.L., Minoshima S., Pagulayan K.F., Li G., Meeker K.D., Kraemer B.C., Petrie E.C. (2016). Repetitive blast exposure in mice and combat veterans causes persistent cerebellar dysfunction. Sci. Transl. Med..

[67] Peskind E.R., Petrie E.C., Cross D.J., Pagulayan K., McCraw K., Hoff D., Hart K., Yu C.E., Raskind M.A., Cook D.G. (2011). Cerebrocerebellar hypometabolism associated with repetitive blast exposure mild traumatic brain injury in 12 Iraq war Veterans with persistent post-concussive symptoms. Neuroimage.

[68] Tate C.M., Wang K.K., Eonta S., Zhang Y., Carr W., Tortella F.C., Hayes R.L., Kamimori G.H. (2013). Serum brain biomarker level, neurocognitive performance, and self-reported symptom changes in soldiers repeatedly exposed to low-level blast: A breacher pilot study. J. Neurotrauma.

[69] Boutté A.M., Thangavelu B., LaValle C.R., Nemes J., Gilsdorf J., Shear D.A., Kamimori G.H. (2019). Brain-related proteins as serum biomarkers of acute, subconcussive blast overpressure exposure: A cohort study of military personnel. PLoS ONE.

[70] Skotak M., LaValle C., Misistia A., Egnoto M.J., Chandra N., Kamimori G. (2019). Occupational Blast Wave Exposure During Multiday 0.50 Caliber Rifle Course. Front. Neurol..

[71] Eonta S.E., Kamimori G.H., Wang K.K.W., Carr W., LaValle C.R., Egnoto M.J., Tate C.M. (2020). Case Study of a Breacher: Investigation of Neurotrauma Biomarker Levels, Self-reported Symptoms, and Functional MRI Analysis Before and After Exposure to Measured Low-Level Blast. Mil. Med..

[72] Wiri S., Massow T., Reid J., Whitty J., Dunbar C., Graves W., Gonzales A., Ortley D., Longwell J., Needham C.E. (2023). Dynamic monitoring of service members to quantify blast exposure levels during combat training using BlackBox Biometrics Blast Gauges: Explosive breaching, shoulder-fired weapons, artillery, mortars, and 0.50 caliber guns. Front. Neurol..

[73] Baker A.J., Topolovec-Vranic J., Michalak A., Pollmann-Mudryj M.A., Ouchterlony D., Cheung B., Tien H.C. (2011). Controlled blast exposure during forced explosive entry training and mild traumatic brain injury. J. Trauma Acute Care Surg..

[74] Thangavelu B., LaValle C.R., Egnoto M.J., Nemes J., Boutté A.M., Kamimori G.H. (2020). Overpressure Exposure from. 50-Caliber Rifle Training Is Associated with Increased Amyloid Beta Peptides in Serum. Front. Neurol..

[75] Agoston D.V., McCullough J., Aniceto R., Lin I.-H., Kamnaksh A., Eklund M., Graves W.M., Dunbar C., Engall J., Schneider E.B. (2022). Blood-Based Biomarkers of repetitive, sub-concussive blast overpressure exposure in the training environment: A pilot study. Neurotrauma Rep..

[76] Roy M.J., Keyser D.O., Rowe S.S., Hernandez R.S., Dovel M., Romero H., Lee D., Menezes M., Magee E., Brooks D.J. (2022). Methodology of the INVestigating traIning assoCiated blasT pAthology (INVICTA) study. BMC Med. Res. Methodol..

[77] McEwen B.S. (2009). The brain is the central organ of stress and adaptation. Neuroimage.

[78] Stone J.R. (2024). Neurological Effects of Repeated Blast Exposure in Special Operations Personnel. J. Neurotrauma.

[79] Cernak I., Kobeissy F.H. (2015). Blast Injuries and Blast-Induced Neurotrauma: Overview of Pathophysiology and Experimental Knowledge Models and Findings. Brain Neurotrauma: Molecular, Neuropsychological, and Rehabilitation Aspects.

[80] Trudeau D.L., Anderson J., Hansen L.M., Shagalov D.N., Schmoller J., Nugent S., Barton S. (1998). Findings of mild traumatic brain injury in combat veterans with PTSD and a history of blast concussion. J. Neuropsychiatry Clin. Neurosci..

[81] Troyanskaya M., Pastorek N.J., Scheibel R.S., Petersen N.J., McCulloch K., Wilde E.A., Henson H.K., Levin H.S. (2015). Combat exposure, PTSD symptoms, and cognition following blast-related traumatic brain injury in OEF/OIF/OND service members and Veterans. Mil. Med..

[82] Perez-Garcia G., Gama Sosa M.A., De Gasperi R., Tschiffely A.E., McCarron R.M., Hof P.R., Gandy S., Ahlers S.T., Elder G.A. (2019). Blast-induced “PTSD”: Evidence from an animal model. Neuropharmacology.

[83] Lange R.T., French L.M., Bailie J.M., Merritt V.C., Pattinson C.L., Hungerford L.D., Lippa S.M., Brickell T.A. (2022). Clinical utility of PTSD, resilience, sleep, and blast as risk factors to predict poor neurobehavioral functioning following traumatic brain injury: A longitudinal study in U.S. military service members. Qual. Life Res..

[84] Agoston D.V., Shutes-David A., Peskind E.R. (2017). Biofluid biomarkers of traumatic brain injury. Brain Inj..

[85] Westrol M.S., Donovan C.M., Kapitanyan R. (2017). Blast Physics and Pathophysiology of Explosive Injuries. Ann. Emerg. Med..

[86] Song H., Cui J., Simonyi A., Johnson C.E., Hubler G.K., DePalma R.G., Gu Z. (2018). Linking blast physics to biological outcomes in mild traumatic brain injury: Narrative review and preliminary report of an open-field blast model. Behav. Brain Res..

[87] Rutter B., Song H., DePalma R.G., Hubler G., Cui J., Gu Z., Johnson C.E. (2021). Shock Wave Physics as Related to Primary Non-Impact Blast-Induced Traumatic Brain Injury. Mil. Med..

[88] Eytan A., Forth S.A., Pickering E.G., Hazael R., Burrows S.J. (2025). Blast wave ingress into a room through an opening—Review of past research and US DoD UFC 3-340-02. Int. J. Prot. Struct..

[89] Ben-Dor G., Igra O., Elperin T., Lifshitz A. (2001). Handbook of Shock Waves.

[90] Scott T.E., Kirkman E., Haque M., Gibb I.E., Mahoney P., Hardman J.G. (2017). Primary blast lung injury—A review. Br. J. Anaesth..

[91] Friedlander F.G. (1946). The diffraction of sound pulses; diffraction by a semi-infinite plane. Proc. R. Soc. Lond. A Math. Phys. Sci..

[92] Ritzel D.V., Parks S.A., Roseveare J., Rude G., Sawyer T.W. Experimental blast simulation for injury studies. Proceedings of the HFM-207 NATO Symposium on a Survey of Blast Injury Across the Full Landscape of Military Science.

[93] Cernak I., Stein D.G., Elder G.A., Ahlers S., Curley K., DePalma R.G., Duda J., Ikonomovic M., Iverson G.L., Kobeissy F. (2017). Preclinical modelling of militarily relevant traumatic brain injuries: Challenges and recommendations for future directions. Brain Inj..

[94] Panzer M.B., Myers B.S., Capehart B.P., Bass C.R. (2012). Development of a finite element model for blast brain injury and the effects of CSF cavitation. Ann. Biomed. Eng..

[95] Goeller J., Wardlaw A., Treichler D., O’Bruba J., Weiss G. (2012). Investigation of cavitation as a possible damage mechanism in blast-induced traumatic brain injury. J. Neurotrauma.

[96] Franck C. (2017). Microcavitation: The key to modeling blast traumatic brain injury?. Concussion.

[97] Cao Y., Risling M., Malm E., Sonden A., Bolling M.F., Skold M.K. (2016). Cellular High-Energy Cavitation Trauma—Description of a Novel In Vitro Trauma Model in Three Different Cell Types. Front. Neurol..

[98] Canchi S., Kelly K., Hong Y., King M.A., Subhash G., Sarntinoranont M. (2017). Controlled single bubble cavitation collapse results in jet-induced injury in brain tissue. J. Mech. Behav. Biomed. Mater..

[99] Adhikari U., Goliaei A., Berkowitz M.L. (2016). Nanobubbles, cavitation, shock waves and traumatic brain injury. Phys. Chem. Chem. Phys..

[100] Li X., Li Z., Wang E., Liang Y., Niu Y., Li Q. (2017). Spectra, energy, and fractal characteristics of blast waves. J. Geophys. Eng..

[101] Loubeau A., Sparrow V.W., Pater L.L., Wright W.M. (2006). High-frequency measurements of blast wave propagation. J. Acoust. Soc. Am..

[102] Cullis I.G. (2001). Blast waves and how they interact with structures. J. R. Army Med. Corps.

[103] Clemedson C.J., Pettersson H. (1956). Propagation of a high explosive air shock wave through different parts of an animal body. Am. J. Physiol..

[104] Clemedson C.J. (1956). Blast injury. Physiol. Rev..

[105] Clemedson C.J. (1956). Shock wave transmission to the central nervous system. Acta Physiol. Scand..

[106] Richmond D.R., Yelverton J.T., Fletcher E.R., Phillips Y.Y. (1989). Physical correlates of eardrum rupture. Ann. Otol. Rhinol. Laryngol. Suppl..

[107] Nakagomi T., Nakano-Doi A., Matsuyama T. (2015). Leptomeninges: A novel stem cell niche harboring ischemia-induced neural progenitors. Histol. Histopathol..

[108] Richmond D.R., Damon E.G., Fletcher E.R., Bowen I.G., White C.S. (1967). The Relationship Between Selected Blast-Wave Parameters and the Response of Mammals Exposed to Air Blast.

[109] Richmond D.R. (1991). Blast criteria for open spaces and enclosures. Scand. Audiol. Suppl..

[110] Jin X., Ma C., Zhang L., Yang K.H., King A.I., Dong G., Zhang J. (2007). Biomechanical response of the bovine pia-arachnoid complex to normal traction loading at varying strain rates. Stapp Car Crash J..

[111] Zhu F., Skelton P., Chou C.C., Mao H., Yang K.H., King A.I. (2013). Biomechanical responses of a pig head under blast loading: A computational simulation. Int. J. Numer. Method. Biomed. Eng..

[112] Courtney A., Courtney M. (2015). The Complexity of Biomechanics Causing Primary Blast-Induced Traumatic Brain Injury: A Review of Potential Mechanisms. Front. Neurol..

[113] Clemedson C.J., Criborn C.O. (1955). Mechanical response of different parts of a living body to a high explosive shock wave impact. Am. J. Physiol..

[114] Prange M.T., Margulies S.S. (2002). Regional, directional, and age-dependent properties of the brain undergoing large deformation. J. Biomech. Eng..

[115] Yu X., Azor A., Sharp D.J., Ghajari M. (2020). Mechanisms of tensile failure of cerebrospinal fluid in blast traumatic brain injury. Extrem. Mech. Lett..

[116] Greiner A., Reiter N., Paulsen F., Holzapfel G.A., Steinmann P., Comellas E., Budday S. (2021). Poro-Viscoelastic Effects During Biomechanical Testing of Human Brain Tissue. Front. Mech. Eng..

[117] Su L., Wang M., Yin J., Ti F., Yang J., Ma C., Liu S., Lu T.J. (2022). Distinguishing poroelasticity and viscoelasticity of brain tissue with time scale. Acta Biomater..

[118] Feng Y., Chen Y., Yao Y., Li X., Zhang A., Genin G.M. (2022). The brain as a structure: A model of how fluid–structure interactions stiffen brain tissue after injury. Eng. Struct..

[119] Greiner A., Reiter N., Hinrichsen J., Kainz M.P., Sommer G., Holzapfel G.A., Steinmann P., Comellas E., Budday S. (2024). Model-driven exploration of poro-viscoelasticity in human brain tissue: Be careful with the parameters!. Interface Focus.

[120] Duck F.A. (1990). Physical Properties of Tissue: A Comprehensive Reference Book.

[121] Morgan M.P.G., Chieng R., Vajuhuudeen Z. Acoustic Impedance. Radiopaedia.org, 2024. https://radiopaedia.org/articles/32118.

[122] Chaussy C., Brendel W., Schmiedt E. (1980). Extracorporeally induced destruction of kidney stones by shock waves. Lancet.

[123] Davros W.J., Garra B.S., Zeman R.K. (1991). Gallstone lithotripsy: Relevant physical principles and technical issues. Radiology.

[124] Namani R., Bayly P.V. (2009). Shear wave propagation in anisotropic soft tissues and gels. Annu. Int. Conf. IEEE Eng. Med. Biol. Soc..

[125] Benzinger T.H. (1950). Physiological Effects of Blast in Air and Water.

[126] Taylor P.A., Ford C.C. (2009). Simulation of blast-induced early-time intracranial wave physics leading to traumatic brain injury. J. Biomech. Eng..

[127] Cernak I. (2014). Blast-induced neurotrauma models and their requirements. Front. Neurol..

[128] Shively S.B., Horkayne-Szakaly I., Jones R.V., Kelly J.P., Armstrong R.C., Perl D.P. (2016). Characterisation of interface astroglial scarring in the human brain after blast exposure: A post-mortem case series. Lancet Neurol..

[129] Haslach H.W., Leahy L.N., Hsieh A.H. (2015). Transient solid-fluid interactions in rat brain tissue under combined translational shear and fixed compression. J. Mech. Behav. Biomed. Mater..

[130] Haslach H.W., Gipple J.M., Leahy L.N. (2017). Influence of high deformation rate, brain region, transverse compression, and specimen size on rat brain shear stress morphology and magnitude. J. Mech. Behav. Biomed. Mater..

[131] Zhang J., Song B., Pintar F.A., Yoganandan N., Chen W., Gennarelli T.A. (2008). How to test brain and brain simulant at ballistic and blast strain rates. Biomed. Sci. Instrum..

[132] Miller S.T., Cooper C.F., Elsbernd P., Kerwin J., Mejia-Alvarez R., Willis A.M. (2021). Localizing Clinical Patterns of Blast Traumatic Brain Injury Through Computational Modeling and Simulation. Front. Neurol..

[133] Kim J.H., Goodrich J.A., Situ R., Rapuano A., Hetherington H., Du F., Parks S., Taylor W., Westmoreland T., Ling G. (2020). Periventricular White Matter Alterations from Explosive Blast in a Large Animal Model: Mild Traumatic Brain Injury or “Subconcussive” Injury?. J. Neuropathol. Exp. Neurol..

[134] Rayleigh L. (1917). On the pressure developed in a liquid during the collapse of a spherical cavity. Philos. Mag..

[135] Adhikari U., Goliaei A., Berkowitz M.L. (2015). Mechanism of membrane poration by shock wave induced nanobubble collapse: A molecular dynamics study. J. Phys. Chem. B.

[136] Fitch M.T., Doller C., Combs C.K., Landreth G.E., Silver J. (1999). Cellular and molecular mechanisms of glial scarring and progressive cavitation: In vivo and in vitro analysis of inflammation-induced secondary injury after CNS trauma. J. Neurosci..

[137] Shuker S.T. (2010). Maxillofacial air-containing cavities, blast implosion injuries, and management. J. Oral Maxillofac. Surg..

[138] Goliaei A., Adhikari U., Berkowitz M.L. (2015). Opening of the blood-brain barrier tight junction due to shock wave induced bubble collapse: A molecular dynamics simulation study. ACS Chem. Neurosci..

[139] Shively S.B., Perl D.P. (2017). Astroglial scarring after blast exposure: Unproven causality—Authors’ reply. Lancet Neurol..

[140] Song H., Konan L.M., Cui J., Johnson C.E., Hubler G.K., DePalma R.G., Gu Z. (2018). Nanometer ultrastructural brain damage following low intensity primary blast wave exposure. Neural Regen. Res..

[141] Konan L.M., Song H., Pentecost G., Fogwe D., Ndam T., Cui J., Johnson C.E., Grant D., White T., Chen M. (2019). Multi-Focal Neuronal Ultrastructural Abnormalities and Synaptic Alterations in Mice after Low-Intensity Blast Exposure. J. Neurotrauma.

[142] Cernak I., Wang Z., Jiang J., Bian X., Savic J. (2001). Ultrastructural and functional characteristics of blast injury-induced neurotrauma. J. Trauma Acute Care Surg..

[143] Stuhmiller J.H. (1997). Biological response to blast overpressure: A summary of modeling. Toxicology.

[144] Moore D.F., Jérusalem A., Nyein M., Noels L., Jaffee M.S., Radovitzky R.A. (2009). Computational biology—Modeling of primary blast effects on the central nervous system. Neuroimage.

[145] Sutar S., Ganpule S. (2022). Evaluation of Blast Simulation Methods for Modeling Blast Wave Interaction with Human Head. J. Biomech. Eng..

[146] Rezaei A., Salimi Jazi M., Karami G., Ziejewski M. (2014). A computational study on brain tissue under blast: Primary and tertiary blast injuries. Int. J. Numer. Method. Biomed. Eng..

[147] VandeVord P.J., Leonardi A.D., Ritzel D. (2016). Bridging the Gap of Standardized Animals Models for Blast Neurotrauma: Methodology for Appropriate Experimental Testing. Methods Mol. Biol..

[148] Panzer M.B., Wood G.W., Bass C.R. (2014). Scaling in neurotrauma: How do we apply animal experiments to people?. Exp. Neurol..

[149] Moss W.C., King M.J., Blackman E.G. (2009). Skull flexure from blast waves: A mechanism for brain injury with implications for helmet design. Phys. Rev. Lett..

[150] Chandra N., Sundaramurthy A. (2016). Primary blast causes mild, moderate, severe and lethal TBI with increasing blast overpressures: Experimental rat injury model. Sci. Rep..

[151] Salzar R.S., Treichler D., Wardlaw A., Weiss G., Goeller J. (2017). Experimental Investigation of Cavitation as a Possible Damage Mechanism in Blast-Induced Traumatic Brain Injury in Post-Mortem Human Subject Heads. J. Neurotrauma.

[152] Kawoos U., Abutarboush R., Gu M., Chen Y., Statz J.K., Goodrich S.Y., Ahlers S.T. (2021). Blast-induced temporal alterations in blood-brain barrier properties in a rodent model. Sci. Rep..

[153] Zhou M., Zhou W., Yang H., Cao L., Li M., Yin P., Zhou Y. (2022). How shockwaves open tight junctions of blood-brain barrier: Comparison of three biomechanical effects. J. Phys. Chem. B.

[154] Op ‘t Eynde J., Yu A.W., Eckersley C.P., Bass C.R. (2020). Primary blast wave protection in combat helmet design: A historical comparison between present day and World War I. PLoS ONE.

[155] Sekine Y., Saitoh D., Yoshimura Y., Fujita M., Araki Y., Kobayashi Y., Kusumi H., Yamagishi S., Suto Y., Tamaki H. (2021). Efficacy of Body Armor in Protection Against Blast Injuries Using a Swine Model in a Confined Space with a Blast Tube. Ann. Biomed. Eng..

[156] Ramasamy A., Hill A.M., Masouros S., Gibb I., Bull A.M., Clasper J.C. (2011). Blast-related fracture patterns: A forensic biomechanical approach. J. R. Soc. Interface.

[157] Yeh D.D., Schecter W.P. (2012). Primary blast injuries--an updated concise review. World J. Surg..

[158] Cernak I. (2010). The importance of systemic response in the pathobiology of blast-induced neurotrauma. Front. Neurol..

[159] McDonald S.J., Sharkey J.M., Sun M., Kaukas L.M., Shultz S.R., Turner R.J., Leonard A.V., Brady R.D., Corrigan F. (2020). Beyond the Brain: Peripheral Interactions after Traumatic Brain Injury. J. Neurotrauma.

[160] Krishnamoorthy V., Komisarow J.M., Laskowitz D.T., Vavilala M.S. (2021). Multiorgan Dysfunction After Severe Traumatic Brain Injury: Epidemiology, Mechanisms, and Clinical Management. Chest.

[161] Sutar S., Ganpule S. (2020). Investigation of wave propagation through head layers with focus on understanding blast wave transmission. Biomech. Model. Mechanobiol..

[162] Simard J.M., Pampori A., Keledjian K., Tosun C., Schwartzbauer G., Ivanova S., Gerzanich V. (2014). Exposure of the thorax to a sublethal blast wave causes a hydrodynamic pulse that leads to perivenular inflammation in the brain. J. Neurotrauma.

[163] Courtney A.C., Courtney M.W. (2009). A thoracic mechanism of mild traumatic brain injury due to blast pressure waves. Med. Hypotheses.

[164] Logsdon A.F., Meabon J.S., Cline M.M., Bullock K.M., Raskind M.A., Peskind E.R., Banks W.A., Cook D.G. (2018). Blast exposure elicits blood-brain barrier disruption and repair mediated by tight junction integrity and nitric oxide dependent processes. Sci. Rep..

[165] Uzunalli G., Herr S., Dieterly A.M., Shi R., Lyle L.T. (2021). Structural disruption of the blood-brain barrier in repetitive primary blast injury. Fluids Barriers CNS.

[166] Goldstein L.E., Fisher A.M., Tagge C.A., Zhang X.L., Velisek L., Sullivan J.A., Upreti C., Kracht J.M., Ericsson M., Wojnarowicz M.W. (2012). Chronic traumatic encephalopathy in blast-exposed military veterans and a blast neurotrauma mouse model. Sci. Transl. Med..

[167] Needham C.E., Ritzel D., Rule G.T., Wiri S., Young L. (2015). Blast Testing Issues and TBI: Experimental Models That Lead to Wrong Conclusions. Front. Neurol..

[168] Kirkman E., Watts S., Cooper G. (2011). Blast injury research models. Philos. Trans. R. Soc. Lond. B Biol. Sci..

[169] Kirkman E., Watts S. (2011). Characterization of the response to primary blast injury. Philos. Trans. R. Soc. Lond. B Biol. Sci..

[170] Ho A.M. (2002). A simple conceptual model of primary pulmonary blast injury. Med. Hypotheses.

[171] van Wessem K.J., Hennus M.P., van Wagenberg L., Koenderman L., Leenen L.P. (2013). Mechanical ventilation increases the inflammatory response induced by lung contusion. J. Surg. Res..

[172] Wang C., Pahk J.B., Balaban C.D., Miller M.C., Wood A.R., Vipperman J.S. (2014). Computational study of human head response to primary blast waves of five levels from three directions. PLoS ONE.

[173] Shuker S.T. (2010). Facial skin-mucosal biodynamic blast injuries and management. J. Oral Maxillofac. Surg..

[174] Nampiaparampil D.E. (2008). Prevalence of chronic pain after traumatic brain injury: A systematic review. JAMA.

[175] Grima N., Ponsford J., Rajaratnam S.M., Mansfield D., Pase M.P. (2016). Sleep Disturbances in Traumatic Brain Injury: A Meta-Analysis. J. Clin. Sleep Med..

[176] Irwin M.R. (2019). Sleep and inflammation: Partners in sickness and in health. Nat. Rev. Immunol..

[177] Louveau A., Smirnov I., Keyes T.J., Eccles J.D., Rouhani S.J., Peske J.D., Derecki N.C., Castle D., Mandell J.W., Lee K.S. (2015). Structural and functional features of central nervous system lymphatic vessels. Nature.

[178] Alves de Lima K., Rustenhoven J., Kipnis J. (2020). Meningeal Immunity and Its Function in Maintenance of the Central Nervous System in Health and Disease. Annu. Rev. Immunol..

[179] Engelhardt B., Coisne C. (2011). Fluids and barriers of the CNS establish immune privilege by confining immune surveillance to a two-walled castle moat surrounding the CNS castle. Fluids Barriers CNS.

[180] Engelhardt B., Carare R.O., Bechmann I., Flugel A., Laman J.D., Weller R.O. (2016). Vascular, glial, and lymphatic immune gateways of the central nervous system. Acta Neuropathol..

[181] Proulx S.T., Engelhardt B. (2022). Central nervous system zoning: How brain barriers establish subdivisions for CNS immune privilege and immune surveillance. J. Intern. Med..

[182] Bolander R., Mathie B., Bir C., Ritzel D., VandeVord P. (2011). Skull flexure as a contributing factor in the mechanism of injury in the rat when exposed to a shock wave. Ann. Biomed. Eng..

[183] Horng S., Therattil A., Moyon S., Gordon A., Kim K., Argaw A.T., Hara Y., Mariani J.N., Sawai S., Flodby P. (2017). Astrocytic tight junctions control inflammatory CNS lesion pathogenesis. J. Clin. Investig..

[184] Savarin C., Stohlman S.A., Atkinson R., Ransohoff R.M., Bergmann C.C. (2010). Monocytes regulate T cell migration through the glia limitans during acute viral encephalitis. J. Virol..

[185] Quagliarello V.J., Wispelwey B., Long W.J., Scheld W.M. (1991). Recombinant human interleukin-1 induces meningitis and blood-brain barrier injury in the rat. Characterization and comparison with tumor necrosis factor. J. Clin. Investig..

[186] Bernier L.P., Brunner C., Cottarelli A., Balbi M. (2021). Location Matters: Navigating Regional Heterogeneity of the Neurovascular Unit. Front. Cell. Neurosci..

[187] Wilhelm I., Nyul-Toth A., Suciu M., Hermenean A., Krizbai I.A. (2016). Heterogeneity of the blood-brain barrier. Tissue Barriers.

[188] Vanlandewijck M., He L., Mäe M.A., Andrae J., Ando K., Del Gaudio F., Nahar K., Lebouvier T., Laviña B., Gouveia L. (2018). A molecular atlas of cell types and zonation in the brain vasculature. Nature.

[189] Tate D.F., York G.E., Reid M.W., Cooper D.B., Jones L., Robin D.A., Kennedy J.E., Lewis J. (2014). Preliminary findings of cortical thickness abnormalities in blast injured service members and their relationship to clinical findings. Brain Imaging Behav..

[190] Han K., Mac Donald C.L., Johnson A.M., Barnes Y., Wierzechowski L., Zonies D., Oh J., Flaherty S., Fang R., Raichle M.E. (2014). Disrupted modular organization of resting-state cortical functional connectivity in U.S. military personnel following concussive ‘mild’ blast-related traumatic brain injury. Neuroimage.

[191] Mac Donald C.L., Johnson A.M., Cooper D., Nelson E.C., Werner N.J., Shimony J.S., Snyder A.Z., Raichle M.E., Witherow J.R., Fang R. (2011). Detection of blast-related traumatic brain injury in U.S. military personnel. N. Engl. J. Med..

[192] Badea A., Kamnaksh A., Anderson R.J., Calabrese E., Long J.B., Agoston D.V. (2018). Repeated mild blast exposure in young adult rats results in dynamic and persistent microstructural changes in the brain. Neuroimage Clin..

[193] Kamnaksh A., Budde M.D., Kovesdi E., Long J.B., Frank J.A., Agoston D.V. (2014). Diffusion tensor imaging reveals acute subcortical changes after mild blast-induced traumatic brain injury. Sci. Rep..

[194] Calabrese E., Du F., Garman R.H., Johnson G.A., Riccio C., Tong L.C., Long J.B. (2014). Diffusion tensor imaging reveals white matter injury in a rat model of repetitive blast-induced traumatic brain injury. J. Neurotrauma.

[195] Gilmore N., Tseng C.J., Maffei C., Tromly S.L., Deary K.B., McKinney I.R., Kelemen J.N., Healy B.C., Hu C.G., Ramos-Llordén G. (2024). Impact of repeated blast exposure on active-duty United States Special Operations Forces. Proc. Natl. Acad. Sci. USA.

[196] Edlow B.L., Tseng C.J., Gilmore N., McKinney I.R., Tromly S.L., Deary K.B., Hu C.G., Healy B.C., Priemer D.S., Mac Donald C.L. (2024). Neuroinflammation at the Gray-White Matter Interface in Active-Duty U.S. Special Operations Forces. Neurotrauma Rep..

[197] Mac Donald C., Johnson A., Cooper D., Malone T., Sorrell J., Shimony J., Parsons M., Snyder A., Raichle M., Fang R. (2013). Cerebellar white matter abnormalities following primary blast injury in US military personnel. PLoS ONE.

[198] Bishop R., Won S.J., Irvine K.A., Basu J., Rome E.S., Swanson R.A. (2022). Blast-induced axonal degeneration in the rat cerebellum in the absence of head movement. Sci. Rep..

[199] Logsdon A.F., Schindler A.G., Meabon J.S., Yagi M., Herbert M.J., Banks W.A., Raskind M.A., Marshall D.A., Keene C.D., Perl D.P. (2020). Nitric oxide synthase mediates cerebellar dysfunction in mice exposed to repetitive blast-induced mild traumatic brain injury. Sci. Rep..

[200] Hayman E., Keledjian K., Stokum J.A., Pampori A., Gerzanich V., Simard J.M. (2018). Selective Vulnerability of the Foramen Magnum in a Rat Blast Traumatic Brain Injury Model. J. Neurotrauma.

[201] Dennis E.L., Rowland J.A., Esopenko C., Tustison N.J., Newsome M.R., Hovenden E.S., Avants B.B., Gill J., Hinds S.R., Kenney K. (2024). Differences in Brain Volume in Military Service Members and Veterans After Blast-Related Mild TBI: A LIMBIC-CENC Study. JAMA Netw. Open.

[202] Petrie E.C., Cross D.J., Yarnykh V.L., Richards T., Martin N.M., Pagulayan K., Hoff D., Hart K., Mayer C., Tarabochia M. (2014). Neuroimaging, behavioral, and psychological sequelae of repetitive combined blast/impact mild traumatic brain injury in Iraq and Afghanistan war veterans. J. Neurotrauma.

[203] Huber B.R., Meabon J.S., Martin T.J., Mourad P.D., Bennett R., Kraemer B.C., Cernak I., Petrie E.C., Emery M.J., Swenson E.R. (2013). Blast exposure causes early and persistent aberrant phospho- and cleaved-tau expression in a murine model of mild blast-induced traumatic brain injury. J. Alzheimer’s Dis..

[204] Schwerin S.C., Chatterjee M., Hutchinson E.B., Djankpa F.T., Armstrong R.C., McCabe J.T., Perl D.P., Juliano S.L. (2021). Expression of GFAP and Tau Following Blast Exposure in the Cerebral Cortex of Ferrets. J. Neuropathol. Exp. Neurol..

[205] Schmahmann J.D., Sherman J.C. (1998). The cerebellar cognitive affective syndrome. Brain.

[206] Ahmadian N., van Baarsen K., van Zandvoort M., Robe P.A. (2019). The Cerebellar Cognitive Affective Syndrome-a Meta-analysis. Cerebellum.

[207] Kheradmand A., Zee D.S. (2011). Cerebellum and ocular motor control. Front. Neurol..

[208] De Zeeuw C.I., Ten Brinke M.M. (2015). Motor Learning and the Cerebellum. Cold Spring Harb. Perspect. Biol..

[209] Chiou L.C., Lee H.J., Ernst M., Huang W.J., Chou J.F., Chen H.L., Mouri A., Chen L.C., Treven M., Mamiya T. (2018). Cerebellar alpha(6)-subunit-containing GABA(A) receptors: A novel therapeutic target for disrupted prepulse inhibition in neuropsychiatric disorders. Br. J. Pharmacol..

[210] Dusick J.R., Wang C., Cohan P., Swerdloff R., Kelly D.F. (2012). Pathophysiology of hypopituitarism in the setting of brain injury. Pituitary.

[211] Molaie A.M., Maguire J. (2018). Neuroendocrine Abnormalities Following Traumatic Brain Injury: An Important Contributor to Neuropsychiatric Sequelae. Front. Endocrinol..

[212] Baxter D., Sharp D.J., Feeney C., Papadopoulou D., Ham T.E., Jilka S., Hellyer P.J., Patel M.C., Bennett A.N., Mistlin A. (2013). Pituitary dysfunction after blast traumatic brain injury: The UK BIOSAP study. Ann. Neurol..

[213] Wilkinson C.W., Pagulayan K.F., Petrie E.C., Mayer C.L., Colasurdo E.A., Shofer J.B., Hart K.L., Hoff D., Tarabochia M.A., Peskind E.R. (2012). High prevalence of chronic pituitary and target-organ hormone abnormalities after blast-related mild traumatic brain injury. Front. Neurol..

[214] Mele C., Pingue V., Caputo M., Zavattaro M., Pagano L., Prodam F., Nardone A., Aimaretti G., Marzullo P. (2021). Neuroinflammation and Hypothalamo-Pituitary Dysfunction: Focus of Traumatic Brain Injury. Int. J. Mol. Sci..

[215] Samdavid Thanapaul R.J.R., Krishnan J.K.S., Govindarajulu M.Y., Pundkar C.Y., Phuyal G., Long J.B., Arun P. (2025). Differential Neuroendocrine Responses and Dysregulation of the Hypothalamic-Pituitary-Adrenal Axis Following Repeated Mild Concussive Impacts and Blast Exposures in a Rat Model. Brain Sci..

[216] Wang F.Q., Wang Q., Wang Y.J., Li Z.M., Li R., Li X.C., Yang L.A., Lu J.W. (2022). Propagation rules of shock waves in confined space under different initial pressure environments. Sci. Rep..

[217] Kelly D.F., McArthur D.L., Levin H., Swimmer S., Dusick J.R., Cohan P., Wang C., Swerdloff R. (2006). Neurobehavioral and quality of life changes associated with growth hormone insufficiency after complicated mild, moderate, or severe traumatic brain injury. J. Neurotrauma.

[218] Prodam F., Gasco V., Caputo M., Zavattaro M., Pagano L., Marzullo P., Belcastro S., Busti A., Perino C., Grottoli S. (2013). Metabolic alterations in patients who develop traumatic brain injury (TBI)-induced hypopituitarism. Growth Horm. IGF Res..

[219] Bondanelli M., Ambrosio M.R., Cavazzini L., Bertocchi A., Zatelli M.C., Carli A., Valle D., Basaglia N., Uberti E.C. (2007). Anterior pituitary function may predict functional and cognitive outcome in patients with traumatic brain injury undergoing rehabilitation. J. Neurotrauma.

[220] Chen Y., O’Shaughnessy T.J., Kamimori G.H., Horner D.M., Egnoto M.J., Bagchi A. (2020). Role of Interfacial Conditions on Blast Overpressure Propagation Into the Brain. Front. Neurol..

[221] Marsh J.L., Bentil S.A. (2021). Cerebrospinal Fluid Cavitation as a Mechanism of Blast-Induced Traumatic Brain Injury: A Review of Current Debates, Methods, and Findings. Front. Neurol..

[222] Hua Y., Lin S., Gu L. (2015). Relevance of Blood Vessel Networks in Blast-Induced Traumatic Brain Injury. Comput. Math. Methods Med..

[223] Saboori P., Sadegh A. (2015). Histology and Morphology of the Brain Subarachnoid Trabeculae. Anat. Res. Int..

[224] Li M., Chen X., Yang Q., Cao S., Wyler S., Yuan R., Zhang L., Liao M., Lv M., Wang F. (2023). Single-nucleus profiling of adult mice sub-ventricular zone after blast-related traumatic brain injury. Sci. Data.

[225] Wimmer I., Tietz S., Nishihara H., Deutsch U., Sallusto F., Gosselet F., Lyck R., Muller W.A., Lassmann H., Engelhardt B. (2019). PECAM-1 Stabilizes Blood-Brain Barrier Integrity and Favors Paracellular T-Cell Diapedesis Across the Blood-Brain Barrier During Neuroinflammation. Front. Immunol..

[226] Wu Y., Liu W., Zhou Y., Hilton T., Zhao Z., Liu W., Wang M., Yeon J., Houck K., Thiagarajan P. (2018). von Willebrand factor enhances microvesicle-induced vascular leakage and coagulopathy in mice with traumatic brain injury. Blood.

[227] de Lanerolle N.C., Bandak F., Kang D., Li A.Y., Du F., Swauger P., Parks S., Ling G., Kim J.H. (2011). Characteristics of an explosive blast-induced brain injury in an experimental model. J. Neuropathol. Exp. Neurol..

[228] Gama Sosa M.A., De Gasperi R., Janssen P.L., Yuk F.J., Anazodo P.C., Pricop P.E., Paulino A.J., Wicinski B., Shaughness M.C., Maudlin-Jeronimo E. (2014). Selective vulnerability of the cerebral vasculature to blast injury in a rat model of mild traumatic brain injury. Acta Neuropathol. Commun..

[229] Szmydynger-Chodobska J., Strazielle N., Zink B.J., Ghersi-Egea J.F., Chodobski A. (2009). The role of the choroid plexus in neutrophil invasion after traumatic brain injury. J. Cereb. Blood Flow Metab..

[230] Johanson C., Stopa E., Baird A., Sharma H. (2011). Traumatic brain injury and recovery mechanisms: Peptide modulation of periventricular neurogenic regions by the choroid plexus-CSF nexus. J. Neural Transm..

[231] Kaur C., Singh J., Lim M.K., Ng B.L., Yap E.P., Ling E.A. (1996). Studies of the choroid plexus and its associated epiplexus cells in the lateral ventricles of rats following an exposure to a single non-penetrative blast. Arch. Histol. Cytol..

[232] Readnower R.D., Chavko M., Adeeb S., Conroy M.D., Pauly J.R., McCarron R.M., Sullivan P.G. (2010). Increase in blood-brain barrier permeability, oxidative stress, and activated microglia in a rat model of blast-induced traumatic brain injury. J. Neurosci. Res..

[233] Bodnar C.N., Watson J.B., Higgins E.K., Quan N., Bachstetter A.D. (2021). Inflammatory Regulation of CNS Barriers After Traumatic Brain Injury: A Tale Directed by Interleukin-1. Front. Immunol..

[234] Solar P., Zamani A., Kubickova L., Dubovy P., Joukal M. (2020). Choroid plexus and the blood-cerebrospinal fluid barrier in disease. Fluids Barriers CNS.

[235] Kant S., Stopa E.G., Johanson C.E., Baird A., Silverberg G.D. (2018). Choroid plexus genes for CSF production and brain homeostasis are altered in Alzheimer’s disease. Fluids Barriers CNS.

[236] Pop V., Badaut J. (2011). A neurovascular perspective for long-term changes after brain trauma. Transl. Stroke Res..

[237] Fenn A.M., Gensel J.C., Huang Y., Popovich P.G., Lifshitz J., Godbout J.P. (2014). Immune activation promotes depression 1 month after diffuse brain injury: A role for primed microglia. Biol. Psychiatry.

[238] Loane D.J., Kumar A. (2016). Microglia in the TBI brain: The good, the bad, and the dysregulated. Exp. Neurol..

[239] Meabon J.S., Schindler A.G., Murray D.R., Colasurdo E.A., Sikkema C.L., Rodriguez J.W., Omer M., Cline M.M., Logsdon A.F., Cross D.J. (2026). Pontine pathology mediates common symptoms of blast-induced chronic mild traumatic brain injury. Brain Commun..

[240] Mondello S., Buki A., Barzo P., Randall J., Provuncher G., Hanlon D., Wilson D., Kobeissy F., Jeromin A. (2014). CSF and plasma amyloid-beta temporal profiles and relationships with neurological status and mortality after severe traumatic brain injury. Sci. Rep..

[241] Blyth B.J., Farhavar A., Gee C., Hawthorn B., He H., Nayak A., Stocklein V., Bazarian J.J. (2009). Validation of serum markers for blood-brain barrier disruption in traumatic brain injury. J. Neurotrauma.

[242] Abbott N.J., Patabendige A.A., Dolman D.E., Yusof S.R., Begley D.J. (2010). Structure and function of the blood-brain barrier. Neurobiol. Dis..

[243] Daneman R., Prat A. (2015). The blood-brain barrier. Cold Spring Harb. Perspect. Biol..

[244] Kadry H., Noorani B., Cucullo L. (2020). A blood-brain barrier overview on structure, function, impairment, and biomarkers of integrity. Fluids Barriers CNS.

[245] Wong A.D., Ye M., Levy A.F., Rothstein J.D., Bergles D.E., Searson P.C. (2013). The blood-brain barrier: An engineering perspective. Front. Neuroeng..

[246] Kaya M., Ahishali B. (2021). Basic physiology of the blood-brain barrier in health and disease: A brief overview. Tissue Barriers.

[247] Wardlaw J.M., Benveniste H., Nedergaard M., Zlokovic B.V., Mestre H., Lee H., Doubal F.N., Brown R., Ramirez J., MacIntosh B.J. (2020). Perivascular spaces in the brain: Anatomy, physiology and pathology. Nat. Rev. Neurol..

[248] Winkler E.A., Kim C.N., Ross J.M., Garcia J.H., Gil E., Oh I., Chen L.Q., Wu D., Catapano J.S., Raygor K. (2022). A single-cell atlas of the normal and malformed human brain vasculature. Science.

[249] McConnell H.L., Kersch C.N., Woltjer R.L., Neuwelt E.A. (2017). The Translational Significance of the Neurovascular Unit. J. Biol. Chem..

[250] Abutarboush R., Gu M., Kawoos U., Mullah S.H., Chen Y., Goodrich S.Y., Lashof-Sullivan M., McCarron R.M., Statz J.K., Bell R.S. (2019). Exposure to Blast Overpressure Impairs Cerebral Microvascular Responses and Alters Vascular and Astrocytic Structure. J. Neurotrauma.

[251] Huber B.R., Meabon J.S., Hoffer Z.S., Zhang J., Hoekstra J.G., Pagulayan K.F., McMillan P.J., Mayer C.L., Banks W.A., Kraemer B.C. (2016). Blast exposure causes dynamic microglial/macrophage responses and microdomains of brain microvessel dysfunction. Neuroscience.

[252] Gama Sosa M.A., De Gasperi R., Perez Garcia G.S., Perez G.M., Searcy C., Vargas D., Spencer A., Janssen P.L., Tschiffely A.E., McCarron R.M. (2019). Low-level blast exposure disrupts gliovascular and neurovascular connections and induces a chronic vascular pathology in rat brain. Acta Neuropathol. Commun..

[253] Crabtree A., McEvoy C., Omer M., Rodriguez J., Charles T., Murray D., Ivory R., Agoston D., Meabon J.S. (2026). Repeated Exposures to Low-Level Blast Induce Acute Region-Specific Changes in the Brain and Acute Elevation in Plasma Cytokine Levels in a Mouse Model. Neurotrauma Rep..

[254] Iadecola C. (2017). The Neurovascular Unit Coming of Age: A Journey through Neurovascular Coupling in Health and Disease. Neuron.

[255] Stamatovic S.M., Johnson A.M., Keep R.F., Andjelkovic A.V. (2016). Junctional proteins of the blood-brain barrier: New insights into function and dysfunction. Tissue Barriers.

[256] Nitta T., Hata M., Gotoh S., Seo Y., Sasaki H., Hashimoto N., Furuse M., Tsukita S. (2003). Size-selective loosening of the blood-brain barrier in claudin-5-deficient mice. J. Cell Biol..

[257] Gavard J., Gutkind J.S. (2008). VE-cadherin and claudin-5: It takes two to tango. Nat. Cell Biol..

[258] Rhind S.G., Shiu M.Y., Tenn C., Nakashima A., Jetly R., Sajja V., Long J.B., Vartanian O. (2025). Repetitive Low-Level Blast Exposure Alters Circulating Myeloperoxidase, Matrix Metalloproteinases, and Neurovascular Endothelial Molecules in Experienced Military Breachers. Int. J. Mol. Sci..

[259] Kuriakose M., Rama Rao K.V., Younger D., Chandra N. (2018). Temporal and Spatial Effects of Blast Overpressure on Blood-Brain Barrier Permeability in Traumatic Brain Injury. Sci. Rep..

[260] Armulik A., Abramsson A., Betsholtz C. (2005). Endothelial/pericyte interactions. Circ. Res..

[261] Hall C.N., Reynell C., Gesslein B., Hamilton N.B., Mishra A., Sutherland B.A., O’Farrell F.M., Buchan A.M., Lauritzen M., Attwell D. (2014). Capillary pericytes regulate cerebral blood flow in health and disease. Nature.

[262] Armulik A., Genové G., Mäe M., Nisancioglu M.H., Wallgard E., Niaudet C., He L., Norlin J., Lindblom P., Strittmatter K. (2010). Pericytes regulate the blood-brain barrier. Nature.

[263] Li C., Chen S., Siedhoff H.R., Grant D., Liu P., Balderrama A., Jackson M., Zuckerman A., Greenlief C.M., Kobeissy F. (2023). Low-intensity open-field blast exposure effects on neurovascular unit ultrastructure in mice. Acta Neuropathol. Commun..

[264] Gama Sosa M.A., De Gasperi R., Pryor D., Perez Garcia G.S., Perez G.M., Abutarboush R., Kawoos U., Hogg S., Ache B., Janssen W.G. (2021). Low-level blast exposure induces chronic vascular remodeling, perivascular astrocytic degeneration and vascular-associated neuroinflammation. Acta Neuropathol. Commun..

[265] Iliff J.J., Wang M., Liao Y., Plogg B.A., Peng W., Gundersen G.A., Benveniste H., Vates G.E., Deane R., Goldman S.A. (2012). A paravascular pathway facilitates CSF flow through the brain parenchyma and the clearance of interstitial solutes, including amyloid beta. Sci. Transl. Med..

[266] Mestre H., Tithof J., Du T., Song W., Peng W., Sweeney A.M., Olveda G., Thomas J.H., Nedergaard M., Kelley D.H. (2018). Flow of cerebrospinal fluid is driven by arterial pulsations and is reduced in hypertension. Nat. Commun..

[267] Xie L., Kang H., Xu Q., Chen M.J., Liao Y., Thiyagarajan M., O’Donnell J., Christensen D.J., Nicholson C., Iliff J.J. (2013). Sleep drives metabolite clearance from the adult brain. Science.

[268] Bojarskaite L., Nafari S., Ravnanger A.K., Frey M.M., Skauli N., Åbjørsbråten K.S., Roth L.C., Amiry-Moghaddam M., Nagelhus E.A., Ottersen O.P. (2024). Role of aquaporin-4 polarization in extracellular solute clearance. Fluids Barriers CNS.

[269] Mestre H., Kostrikov S., Mehta R.I., Nedergaard M. (2017). Perivascular spaces, glymphatic dysfunction, and small vessel disease. Clin. Sci..

[270] Piantino J., Schwartz D.L., Luther M., Newgard C., Silbert L., Raskind M., Pagulayan K., Kleinhans N., Iliff J., Peskind E. (2021). Link between Mild Traumatic Brain Injury, Poor Sleep, and Magnetic Resonance Imaging: Visible Perivascular Spaces in Veterans. J. Neurotrauma.

[271] Braun M., Sevao M., Keil S.A., Gino E., Wang M.X., Lee J., Haveliwala M.A., Klein E., Agarwal S., Pedersen T. (2024). Macroscopic changes in aquaporin-4 underlie blast traumatic brain injury-related impairment in glymphatic function. Brain.

[272] Iliff J.J., Chen M.J., Plog B.A., Zeppenfeld D.M., Soltero M., Yang L., Singh I., Deane R., Nedergaard M. (2014). Impairment of glymphatic pathway function promotes tau pathology after traumatic brain injury. J. Neurosci..

[273] Ren Z., Iliff J.J., Yang L., Yang J., Chen X., Chen M.J., Giese R.N., Wang B., Shi X., Nedergaard M. (2013). ‘Hit & Run’ model of closed-skull traumatic brain injury (TBI) reveals complex patterns of post-traumatic AQP4 dysregulation. J. Cereb. Blood Flow Metab..

[274] Smith D.H., Meaney D.F., Shull W.H. (2003). Diffuse axonal injury in head trauma. J. Head Trauma Rehabil..

[275] Logsdon A.F., Lucke-Wold B.P., Turner R.C., Huber J.D., Rosen C.L., Simpkins J.W. (2015). Role of Microvascular Disruption in Brain Damage from Traumatic Brain Injury. Compr. Physiol..

[276] Kallakuri S., Desai A., Feng K., Tummala S., Saif T., Chen C., Zhang L., Cavanaugh J.M., King A.I. (2017). Neuronal Injury and Glial Changes Are Hallmarks of Open Field Blast Exposure in Swine Frontal Lobe. PLoS ONE.

[277] Kovacs S.K., Leonessa F., Ling G.S. (2014). Blast TBI Models, Neuropathology, and Implications for Seizure Risk. Front. Neurol..

[278] Frati A., Cerretani D., Fiaschi A.I., Frati P., Gatto V., La Russa R., Pesce A., Pinchi E., Santurro A., Fraschetti F. (2017). Diffuse Axonal Injury and Oxidative Stress: A Comprehensive Review. Int. J. Mol. Sci..

[279] Lozano D., Gonzales-Portillo G.S., Acosta S., de la Pena I., Tajiri N., Kaneko Y., Borlongan C.V. (2015). Neuroinflammatory responses to traumatic brain injury: Etiology, clinical consequences, and therapeutic opportunities. Neuropsychiatr. Dis. Treat..

[280] Baracaldo-Santamaria D., Ariza-Salamanca D.F., Corrales-Hernandez M.G., Pachon-Londono M.J., Hernandez-Duarte I., Calderon-Ospina C.A. (2022). Revisiting Excitotoxicity in Traumatic Brain Injury: From Bench to Bedside. Pharmaceutics.

[281] Przekwas A., Somayaji M.R., Gupta R.K. (2016). Synaptic Mechanisms of Blast-Induced Brain Injury. Front. Neurol..

[282] Smith M., Piehler T., Benjamin R., Farizatto K.L., Pait M.C., Almeida M.F., Ghukasyan V.V., Bahr B.A. (2016). Blast waves from detonated military explosive reduce GluR1 and synaptophysin levels in hippocampal slice cultures. Exp. Neurol..

[283] Gama Sosa M.A., De Gasperi R., Pryor D., Perez Garcia G.S., Perez G.M., Abutarboush R., Kawoos U., Hogg S., Ache B., Sowa A. (2023). Late chronic local inflammation, synaptic alterations, vascular remodeling and arteriovenous malformations in the brains of male rats exposed to repetitive low-level blast overpressures. Acta Neuropathol. Commun..

[284] Medaglia J.D. (2017). Functional Neuroimaging in Traumatic Brain Injury: From Nodes to Networks. Front. Neurol..

[285] Irimia A., Wang B., Aylward S.R., Prastawa M.W., Pace D.F., Gerig G., Hovda D.A., Kikinis R., Vespa P.M., Van Horn J.D. (2012). Neuroimaging of structural pathology and connectomics in traumatic brain injury: Toward personalized outcome prediction. Neuroimage Clin..

[286] Qin D., Wang J., Le A., Wang T.J., Chen X., Wang J. (2021). Traumatic Brain Injury: Ultrastructural Features in Neuronal Ferroptosis, Glial Cell Activation and Polarization, and Blood-Brain Barrier Breakdown. Cells.

[287] Rowland J.A., Martindale S.L. (2024). Considerations for the assessment of blast exposure in service members and veterans. Front. Neurol..

[288] Turner S.M., Sloley S.S., Bailie J.M., Babakhanyan I., Gregory E. (2022). Perspectives on Development of Measures to Estimate Career Blast Exposure History in Service Members and Veterans. Front. Neurol..

[289] Goldstein L.E., McKee A.C., Stanton P.K. (2014). Considerations for animal models of blast-related traumatic brain injury and chronic traumatic encephalopathy. Alzheimer’s Res. Ther..

[290] Elder G.A., Gama Sosa M.A., De Gasperi R., Stone J.R., Dickstein D.L., Haghighi F., Hof P.R., Ahlers S.T. (2015). Vascular and inflammatory factors in the pathophysiology of blast-induced brain injury. Front. Neurol..

[291] Elder G.A., Gama Sosa M.A., De Gasperi R., Perez Garcia G., Perez G.M., Abutarboush R., Kawoos U., Zhu C.W., Janssen W.G.M., Stone J.R. (2024). The Neurovascular Unit as a Locus of Injury in Low-Level Blast-Induced Neurotrauma. Int. J. Mol. Sci..

[292] Hablitz L.M., Plá V., Giannetto M., Vinitsky H.S., Stæger F.F., Metcalfe T., Nguyen R., Benrais A., Nedergaard M. (2020). Circadian control of brain glymphatic and lymphatic fluid flow. Nat. Commun..

[293] Konig S., Jayarajan V., Wray S., Kamm R., Moeendarbary E. (2025). Mechanobiology of the blood-brain barrier during development, disease and ageing. Nat. Commun..

[294] Wu Y., Wu H., Guo X., Pluimer B., Zhao Z. (2020). Blood-Brain Barrier Dysfunction in Mild Traumatic Brain Injury: Evidence from Preclinical Murine Models. Front. Physiol..

[295] Cheng J., Gu J., Ma Y., Yang T., Kuang Y., Li B., Kang J. (2010). Development of a rat model for studying blast-induced traumatic brain injury. J. Neurol. Sci..

[296] Sosa M.A., De Gasperi R., Paulino A.J., Pricop P.E., Shaughness M.C., Maudlin-Jeronimo E., Hall A.A., Janssen W.G., Yuk F.J., Dorr N.P. (2013). Blast overpressure induces shear-related injuries in the brain of rats exposed to a mild traumatic brain injury. Acta Neuropathol. Commun..

[297] Lotan E., Morley C., Newman J., Qian M., Abu-Amara D., Marmar C., Lui Y.W. (2018). Prevalence of Cerebral Microhemorrhage following Chronic Blast-Related Mild Traumatic Brain Injury in Military Service Members Using Susceptibility-Weighted MRI. AJNR Am. J. Neuroradiol..

[298] Kilgore M.O., Hubbard W.B. (2024). Effects of Low-Level Blast on Neurovascular Health and Cerebral Blood Flow: Current Findings and Future Opportunities in Neuroimaging. Int. J. Mol. Sci..

[299] Conway D.E., Breckenridge M.T., Hinde E., Gratton E., Chen C.S., Schwartz M.A. (2013). Fluid shear stress on endothelial cells modulates mechanical tension across VE-cadherin and PECAM-1. Curr. Biol..

[300] Cucullo L., Hossain M., Puvenna V., Marchi N., Janigro D. (2011). The role of shear stress in Blood-Brain Barrier endothelial physiology. BMC Neurosci..

[301] Zheng Q., Zou Y., Teng P., Chen Z., Wu Y., Dai X., Li X., Hu Z., Wu S., Xu Y. (2022). Mechanosensitive Channel PIEZO1 Senses Shear Force to Induce KLF2/4 Expression via CaMKII/MEKK3/ERK5 Axis in Endothelial Cells. Cells.

[302] Chien S. (2008). Effects of disturbed flow on endothelial cells. Ann. Biomed. Eng..

[303] Noria S., Cowan D.B., Gotlieb A.I., Langille B.L. (1999). Transient and steady-state effects of shear stress on endothelial cell adherens junctions. Circ. Res..

[304] Predescu S.A., Predescu D.N., Malik A.B. (2007). Molecular determinants of endothelial transcytosis and their role in endothelial permeability. Am. J. Physiol. Lung Cell. Mol. Physiol..

[305] Chen B.R., Kozberg M.G., Bouchard M.B., Shaik M.A., Hillman E.M. (2014). A critical role for the vascular endothelium in functional neurovascular coupling in the brain. J. Am. Heart Assoc..

[306] Kisler K., Nelson A.R., Rege S.V., Ramanathan A., Wang Y., Ahuja A., Lazic D., Tsai P.S., Zhao Z., Zhou Y. (2017). Pericyte degeneration leads to neurovascular uncoupling and limits oxygen supply to brain. Nat. Neurosci..

[307] Kisler K., Nelson A.R., Montagne A., Zlokovic B.V. (2017). Cerebral blood flow regulation and neurovascular dysfunction in Alzheimer disease. Nat. Rev. Neurosci..

[308] Muneer P.M.A., Schuetz H., Wang F., Skotak M., Jones J., Gorantla S., Zimmerman M.C., Chandra N., Haorah J. (2013). Induction of oxidative and nitrosative damage leads to cerebrovascular inflammation in an animal model of mild traumatic brain injury induced by primary blast. Free Radic. Biol. Med..

[309] Lucke-Wold B.P., Logsdon A.F., Smith K.E., Turner R.C., Alkon D.L., Tan Z., Naser Z.J., Knotts C.M., Huber J.D., Rosen C.L. (2015). Bryostatin-1 Restores Blood Brain Barrier Integrity following Blast-Induced Traumatic Brain Injury. Mol. Neurobiol..

[310] Shetty A.K., Mishra V., Kodali M., Hattiangady B. (2014). Blood brain barrier dysfunction and delayed neurological deficits in mild traumatic brain injury induced by blast shock waves. Front. Cell. Neurosci..

[311] Vita S.M., Redell J.B., Maynard M.E., Zhao J., Grill R.J., Dash P.K., Grayson B.E. (2020). P-glycoprotein Expression Is Upregulated in a Pre-Clinical Model of Traumatic Brain Injury. Neurotrauma Rep..

[312] Chodobski A., Zink B.J., Szmydynger-Chodobska J. (2011). Blood-brain barrier pathophysiology in traumatic brain injury. Transl. Stroke Res..

[313] Sweeney M.D., Zhao Z., Montagne A., Nelson A.R., Zlokovic B.V. (2019). Blood-Brain Barrier: From Physiology to Disease and Back. Physiol. Rev..

[314] Haruwaka K., Ikegami A., Tachibana Y., Ohno N., Konishi H., Hashimoto A., Matsumoto M., Kato D., Ono R., Kiyama H. (2019). Dual microglia effects on blood brain barrier permeability induced by systemic inflammation. Nat. Commun..

[315] Spear A.M., Davies E.M., Taylor C., Whiting R., Macildowie S., Kirkman E., Midwinter M., Watts S.A. (2015). Blast Wave Exposure to the Extremities Causes Endothelial Activation and Damage. Shock.

[316] Hubbard W.B., Dong J.F., Cruz M.A., Rumbaut R.E. (2021). Links between thrombosis and inflammation in traumatic brain injury. Thromb. Res..

[317] Villalba N., Sackheim A.M., Nunez I.A., Hill-Eubanks D.C., Nelson M.T., Wellman G.C., Freeman K. (2017). Traumatic Brain Injury Causes Endothelial Dysfunction in the Systemic Microcirculation through Arginase-1-Dependent Uncoupling of Endothelial Nitric Oxide Synthase. J. Neurotrauma.

[318] Hubbard W.B., Sim M.M.S., Saatman K.E., Sullivan P.G., Wood J.P. (2022). Tissue factor release following traumatic brain injury drives thrombin generation. Res. Pract. Thromb. Haemost..

[319] Cohen M.J., Brohi K., Ganter M.T., Manley G.T., Mackersie R.C., Pittet J.F. (2007). Early coagulopathy after traumatic brain injury: The role of hypoperfusion and the protein C pathway. J. Trauma..

[320] Condron M., Rowell S., Dewey E., Anderson T., Lealiiee L., Farrell D., Hinson H. (2018). The procoagulant molecule plasminogen activator inhibitor-1 is associated with injury severity and shock in patients with and without traumatic brain injury. J. Trauma. Acute Care Surg..

[321] Villalba N., Sonkusare S.K., Longden T.A., Tran T.L., Sackheim A.M., Nelson M.T., Wellman G.C., Freeman K. (2014). Traumatic brain injury disrupts cerebrovascular tone through endothelial inducible nitric oxide synthase expression and nitric oxide gain of function. J. Am. Heart Assoc..

[322] Kuriakose M., Younger D., Ravula A.R., Alay E., Rama Rao K.V., Chandra N. (2019). Synergistic Role of Oxidative Stress and Blood-Brain Barrier Permeability as Injury Mechanisms in the Acute Pathophysiology of Blast-induced Neurotrauma. Sci. Rep..

[323] Bayir H., Kagan V.E., Tyurina Y.Y., Tyurin V., Ruppel R.A., Adelson P.D., Graham S.H., Janesko K., Clark R.S., Kochanek P.M. (2002). Assessment of antioxidant reserves and oxidative stress in cerebrospinal fluid after severe traumatic brain injury in infants and children. Pediatr. Res..

[324] Arun P., Abu-Taleb R., Oguntayo S., Wang Y., Valiyaveettil M., Long J.B., Nambiar M.P. (2013). Acute mitochondrial dysfunction after blast exposure: Potential role of mitochondrial glutamate oxaloacetate transaminase. J. Neurotrauma.

[325] Hubbard W.B., Vekaria H.J., Velmurugan G.V., Kalimon O.J., Prajapati P., Brown E., Geisler J.G., Sullivan P.G. (2023). Mitochondrial Dysfunction After Repeated Mild Blast Traumatic Brain Injury Is Attenuated by a Mild Mitochondrial Uncoupling Prodrug. J. Neurotrauma.

[326] Lamade A.M., Anthonymuthu T.S., Hier Z.E., Gao Y., Kagan V.E., Bayir H. (2020). Mitochondrial damage & lipid signaling in traumatic brain injury. Exp. Neurol..

[327] Hang C.H., Shi J.X., Li J.S., Wu W., Yin H.X. (2005). Concomitant upregulation of nuclear factor-kB activity, proinflammatory cytokines and ICAM-1 in the injured brain after cortical contusion trauma in a rat model. Neurol. India.

[328] Carlos T.M., Clark R.S., Franicola-Higgins D., Schiding J.K., Kochanek P.M. (1997). Expression of endothelial adhesion molecules and recruitment of neutrophils after traumatic brain injury in rats. J. Leukoc. Biol..

[329] Mussbacher M., Salzmann M., Brostjan C., Hoesel B., Schoergenhofer C., Datler H., Hohensinner P., Basilio J., Petzelbauer P., Assinger A. (2019). Cell Type-Specific Roles of NF-kappaB Linking Inflammation and Thrombosis. Front. Immunol..

[330] Semple B.D., Bye N., Rancan M., Ziebell J.M., Morganti-Kossmann M.C. (2010). Role of CCL2 (MCP-1) in traumatic brain injury (TBI): Evidence from severe TBI patients and CCL2−/− mice. J. Cereb. Blood Flow Metab..

[331] Das M., Mohapatra S., Mohapatra S.S. (2012). New perspectives on central and peripheral immune responses to acute traumatic brain injury. J. Neuroinflammation.

[332] Wang K.Y., Yu G.F., Zhang Z.Y., Huang Q., Dong X.Q. (2012). Plasma high-mobility group box 1 levels and prediction of outcome in patients with traumatic brain injury. Clin. Chim. Acta.

[333] Kim S.Y., Son M., Lee S.E., Park I.H., Kwak M.S., Han M., Lee H.S., Kim E.S., Kim J.Y., Lee J.E. (2018). High-Mobility Group Box 1-Induced Complement Activation Causes Sterile Inflammation. Front. Immunol..

[334] Thurman J.M., Renner B. (2011). Dynamic control of the complement system by modulated expression of regulatory proteins. Lab. Investig..

[335] Karve I.P., Taylor J.M., Crack P.J. (2016). The contribution of astrocytes and microglia to traumatic brain injury. Br. J. Pharmacol..

[336] Loane D.J., Kumar A., Stoica B.A., Cabatbat R., Faden A.I. (2014). Progressive neurodegeneration after experimental brain trauma: Association with chronic microglial activation. J. Neuropathol. Exp. Neurol..

[337] Ramlackhansingh A.F., Brooks D.J., Greenwood R.J., Bose S.K., Turkheimer F.E., Kinnunen K.M., Gentleman S., Heckemann R.A., Gunanayagam K., Gelosa G. (2011). Inflammation after trauma: Microglial activation and traumatic brain injury. Ann. Neurol..

[338] Sullivan D.R., Miller M.W., Wolf E.J., Logue M.W., Robinson M.E., Fortier C.B., Fonda J.R., Wang D.J., Milberg W.P., McGlinchey R.E. (2021). Cerebral perfusion is associated with blast exposure in military personnel without moderate or severe TBI. J. Cereb. Blood Flow Metab..

[339] Sandsmark D.K., Elliott J.E., Lim M.M. (2017). Sleep-Wake Disturbances After Traumatic Brain Injury: Synthesis of Human and Animal Studies. Sleep.

[340] Wickwire E.M., Schnyer D.M., Germain A., Williams S.G., Lettieri C.J., McKeon A.B., Scharf S.M., Stocker R., Albrecht J., Badjatia N. (2018). Sleep, Sleep Disorders, and Circadian Health following Mild Traumatic Brain Injury in Adults: Review and Research Agenda. J. Neurotrauma.

[341] Lu J., Goh S.J., Tng P.Y., Deng Y.Y., Ling E.A., Moochhala S. (2009). Systemic inflammatory response following acute traumatic brain injury. Front. Biosci..

[342] Zetterberg H., Smith D.H., Blennow K. (2013). Biomarkers of mild traumatic brain injury in cerebrospinal fluid and blood. Nat. Rev. Neurol..

[343] Bouras M., Asehnoune K., Roquilly A. (2022). Immune modulation after traumatic brain injury. Front. Med..

[344] Scott M.C., Bedi S.S., Olson S.D., Sears C.M., Cox C.S. (2021). Microglia as therapeutic targets after neurological injury: Strategy for cell therapy. Expert Opin. Ther. Targets.

[345] Ning Y.L., Zhou Y.G. (2015). Shock tubes and blast injury modeling. Chin. J. Traumatol..

[346] Hajiaghamemar M., Seidi M., Oeur R.A., Margulies S.S. (2019). Toward development of clinically translatable diagnostic and prognostic metrics of traumatic brain injury using animal models: A review and a look forward. Exp. Neurol..

[347] LaPlaca M.C., Huie J.R., Alam H.B., Bachstetter A.D., Bayir H., Bellgowan P.F., Cummings D., Dixon C.E., Ferguson A.R., Ferland-Beckham C. (2021). Pre-Clinical Common Data Elements for Traumatic Brain Injury Research: Progress and Use Cases. J. Neurotrauma.

[348] Meeuws S., Yue J.K., Huijben J.A., Nair N., Lingsma H.F., Bell M.J., Manley G.T., Maas A.I.R. (2020). Common Data Elements: Critical Assessment of Harmonization between Current Multi-Center Traumatic Brain Injury Studies. J. Neurotrauma.

[349] Iboaya A., Harris J.L., Arickx A.N., Nudo R.J. (2019). Models of Traumatic Brain Injury in Aged Animals: A Clinical Perspective. Neurorehabilit. Neural Repair.

[350] Eagle S.R., Puccio A.M., Nelson L.D., McCrea M., Giacino J., Diaz-Arrastia R., Conkright W., Jain S., Sun X., Manley G. (2023). Association of obesity with mild traumatic brain injury symptoms, inflammatory profile, quality of life and functional outcomes: A TRACK-TBI Study. J. Neurol. Neurosurg. Psychiatry.

[351] McFadyen C.A., Zeiler F.A., Newcombe V., Synnot A., Steyerberg E., Gruen R.L., Rosand J., Palotie A., Maas A.I.R., Menon D.K. (2021). Apolipoprotein E4 Polymorphism and Outcomes from Traumatic Brain Injury: A Living Systematic Review and Meta-Analysis. J. Neurotrauma.

[352] Rabinowitz A.R., Li X., McCauley S.R., Wilde E.A., Barnes A., Hanten G., Mendez D., McCarthy J.J., Levin H.S. (2015). Prevalence and Predictors of Poor Recovery from Mild Traumatic Brain Injury. J. Neurotrauma.

[353] Nelson L.D., Temkin N.R., Dikmen S., Barber J., Giacino J.T., Yuh E., Levin H.S., McCrea M.A., Stein M.B., Mukherjee P. (2019). Recovery After Mild Traumatic Brain Injury in Patients Presenting to US Level I Trauma Centers: A Transforming Research and Clinical Knowledge in Traumatic Brain Injury (TRACK-TBI) Study. JAMA Neurol..

[354] Carr W., Polejaeva E., Grome A., Crandall B., LaValle C., Eonta S.E., Young L.A. (2015). Relation of repeated low-level blast exposure with symptomology similar to concussion. J. Head Trauma Rehabil..

[355] Jean A., Nyein M.K., Zheng J.Q., Moore D.F., Joannopoulos J.D., Radovitzky R. (2014). An animal-to-human scaling law for blast-induced traumatic brain injury risk assessment. Proc. Natl. Acad. Sci. USA.

[356] Saiki T., Shimada K., Ishijima A., Song H., Qi X., Okamoto Y., Mizushima A., Mita Y., Hosobata T., Takeda M. (2023). Single-shot optical imaging with spectrum circuit bridging timescales in high-speed photography. Sci. Adv..

[357] Dal Cengio Leonardi A., Keane N.J., Bir C.A., Ryan A.G., Xu L., Vandevord P.J. (2012). Head orientation affects the intracranial pressure response resulting from shock wave loading in the rat. J. Biomech..

[358] Hua Y., Wang Y., Gu L. (2017). Primary blast waves induced brain dynamics influenced by head orientations. Biomed. Eng. Lett..

[359] Zhang L., Makwana R., Sharma S. (2013). Brain response to primary blast wave using validated finite element models of human head and advanced combat helmet. Front. Neurol..

